# Peripheral T cell cytotoxicity predicts T cell function in the tumor microenvironment

**DOI:** 10.1038/s41598-019-39345-5

**Published:** 2019-02-22

**Authors:** Kota Iwahori, Yasushi Shintani, Soichiro Funaki, Yoko Yamamoto, Mitsunobu Matsumoto, Tetsuya Yoshida, Akiko Morimoto-Okazawa, Atsunari Kawashima, Eiichi Sato, Stephen Gottschalk, Meinoshin Okumura, Atsushi Kumanogoh, Hisashi Wada

**Affiliations:** 1 Department of Clinical Research in Tumor Immunology, Graduate School of Medicine, Osaka University, Suita, Osaka Japan; 2 Department of Respiratory Medicine and Clinical Immunology, Graduate School of Medicine, Osaka University, Suita, Osaka Japan; 3 Department of General Thoracic Surgery, Graduate School of Medicine, Osaka University, Suita, Osaka Japan; 4 Shionogi & Co., Ltd., Toyonaka, Osaka Japan; 5 Department of Frontier Research in Tumor Immunology, Graduate School of Medicine, Osaka University, Suita, Osaka Japan; 6 Department of Pathology (Medical Research Center), Institute of Medical Science, Tokyo Medical University, Tokyo, Japan; 7 Department of Bone Marrow Transplant and Cellular Therapy, St. Jude Children’s Research Hospital, Memphis, TN USA

## Abstract

Cancer immunotherapy, including immune checkpoint inhibitors, exerts beneficial effects in cancer patients. However, immune checkpoint inhibitors are only advantageous for a limited population of cancer patients. Therefore, companion diagnostics are needed in order to identify patients for whom these therapies are effective. In the present study, we evaluated detailed immunological aspects in clinical specimens from non-small cell lung cancer (NSCLC) patients. We analyzed the immune profiles, T cell cytotoxicity, and TCR repertoire of peripheral blood, normal lung tissue, and tumor tissue from NSCLC patients. By using bispecific T-cell engager technology to assess the cytotoxicity of T cells, we found that the cytotoxicity of tumor-infiltrated T cells closely correlated with that of peripheral T cells. This correlation was supported by the immune profiles, cytokine production, and results of the TCR repertoire analysis from these specimens. We also found that the cytotoxicity of peripheral T cells has potential as a predictor of the effects of nivolumab in the tumor microenvironment. These results imply further applications to blood-based immune monitoring systems and predictive biomarkers for cancer immunotherapy.

## Introduction

Immune checkpoint inhibitors open a new era for cancer immunotherapy. The anti-PD-1 blocking antibody exerts beneficial effects in a limited population of cancer patients^1^. PD-L1 staining has been developed for companion diagnostics to this treatment^2,3^. Clinical trials for novel immune checkpoint inhibitors are ongoing and effective companion diagnostics for these therapeutics are a critical focus worldwide^4^. A clearer understanding of the tumor immune microenvironment is needed for the development of new therapeutic targets and companion diagnostics for cancer immunotherapy, with the identification of tumor antigen-specific T cells in tumor tissue representing a critical issue. However, evaluations of the activities of tumor antigen-specific T cells are challenging, particularly in cancer patients.

Tumor antigen-specific T cells exhibit cytotoxic activity against tumor cells during the antitumor immune response. The anti-PD-1 blocking antibody is estimated to enhance tumor antigen-specific T cell activity^5^. On the other hand, chimeric antigen receptor T cells (CAR-T cells) and bispecific T-cell engager (BiTE) redirect T cells to tumor cells^6^. BiTE consists of two single chain variable fragments (scFVs) connected by a short linker, which are specific for CD3 expressed on T cells and an antigen expressed on the surface of tumor cells. The pattern of T cell cytotoxicity induced by BiTE shows some similarities to tumor cell killing by endogenous tumor antigen-specific T cells^7,8^. In the present study, we evaluated the cytotoxic activity of T cells in freshly isolated tumor tissues from non-small cell lung cancer (NSCLC) patients using BiTE technology.

Since the population of cancer patients for whom immune checkpoint inhibitors are beneficial is limited, the development of companion diagnostics is urgently needed. Regarding the anti-PD-1 blocking antibody, PD-L1 staining in tumor cells is applied in clinical practice. Other than tissue biopsies, attempts to develop diagnostic procedures using peripheral blood samples are one of the focuses for companion diagnostics with cancer immunotherapy. In animal experiments, IFNγ production by peripheral lymphocytes was shown to predict the survival of tumor-bearing mice receiving the dual PD-1/CTLA-4 blockade^9^. In melanoma patients, neoantigen- and shared antigen-specific T cells have both been identified in the circulating PD-1^+^/CD8^+^ T cell population. Moreover, a clonal overlap exists between these cells in blood and tumor-infiltrating T cells^10^. In the present study, we evaluated the cytotoxic activity of T cells in tumor tissues and analyzed their relationship with peripheral blood T cells as a step towards the development of companion diagnostics using blood samples for cancer immunotherapy.

## Results

### Immune profiling of NSCLC patients

**Figure 1:**
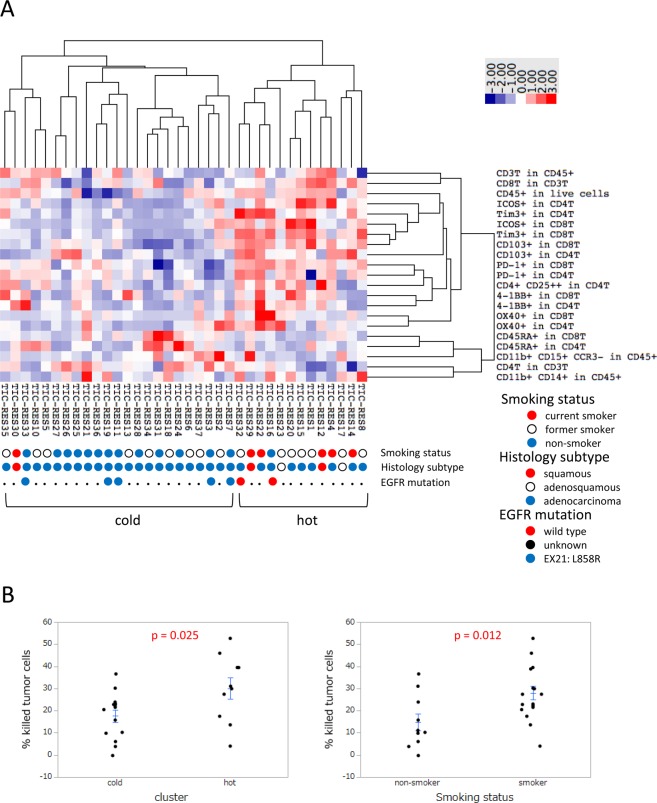
Immune profiling of NSCLC tumor tissues. (**A**) Cluster analysis for the immune profiling of tumor-infiltrating cells (TIC) from NSCLC patients (n = 36). A hierarchical clustering algorithm was applied using the uncentered correlation coefficient as a measure of similarity and the method of average linkage by Cluster 3.0 and TreeView software. Individual data were transformed to Z scores for standardization purposes. Immune parameters measured by flow cytometry are listed. Clinical characteristics, including the smoking status, histology subtype, and EGFR mutation status, were shown for each patient. (**B**) Cytotoxic activity of T cells from lung tumor tissues. Regarding immune clustering (n = 24) and the smoking status (n = 27), the cytotoxic activity of tumor-infiltrating T cells was evaluated in a 48-hour co-culture of U251 (E/T ratio of 5:1) with EphA2-BiTE (100 ng/ml). Each dot represents one patient. Data were combined from triplicate experiments. The mean ± SEM was shown. An unpaired two-tailed Student’s *t*-test was used to examine the significance of differences between samples, with a p value < 0.05 indicating a significant difference.

As the basis for understanding immune responses in the tumor microenvironment, we analyzed the immune profiles of peripheral blood, normal lung tissues, and lung tumor tissues from NSCLC patients (Supplementary Table S1). Based on flow cytometric data, we performed a cluster analysis of immune profiles. A heat map of lung tumor tissues showed two clearly separated clusters, which consisted of an immunologically “hot” cluster and immunologically “cold” cluster (Fig. 1A). Although the heat maps of peripheral blood and normal lung tissues showed different patterns from that of lung tumor tissues, each profile between them partially correlated with each other (Supplementary Figs S1 and S2).

In order to extract the clinical features of the two clusters, we evaluated the smoking status, histology subtype, and EGFR mutation status among these clusters (Fig. 1A). Thirteen out of 14 non-smokers (92.9%) belonged to the “cold” cluster. Four out of five squamous or adenosquamous cell carcinomas (80.0%) were in the “hot” cluster. Regarding the EGFR mutation status, five patients with an EGFR mutation (EX21: L858R) (100%) belonged to the “cold” cluster. Although no significant differences were observed in tumor volumes between the two clusters, the SUVmax of lung tumors on FDG-PET was higher for the “hot” cluster than for the “cold” cluster (Supplementary Fig. S3).

### Cytotoxic activity of T cells

One of the critical factors for immune responses in the tumor microenvironment is the cytotoxic activity of tumor antigen-specific T cells. In order to evaluate the cytotoxic activity of T cells in fresh lung tumor tissue, we developed an assay system using BiTE (Supplementary Fig. S4). In this assay, we used BiTE specific for EphA2 and CD3. Although PBMC with CD19/CD3 BiTE did not exhibit cytolytic activity in CD19-negative tumor cell lines (U251, RERF, and A549), PBMC with EphA2/CD3 BiTE was cytotoxic to these cells expressing EphA2, particularly EphA2 high-expressing U251 cells (Supplementary Fig. S4A,B). Furthermore, IFNγ production with EphA2/CD3 BiTE increased in the co-culture of PBMC and EphA2-expressing U251 cells, but not EphA2-negative BALL-1 cells (Supplementary Fig. S4C). In the analysis of the limited number of cells obtained from lung tumor tissues, we optimized co-culture conditions using U251 cells, which were the most sensitive to EphA2/CD3 BiTE-mediated killing. Cytotoxicity to U251 cells was assessed under different effector to target ratios, BiTE concentrations, and co-culture periods (Supplementary Fig. S4D). In addition to cytotoxicity, we examined IFNγ secretion from CD4^+^ and CD8^+^ T cells under different co-culture periods (Supplementary Fig. S4E). Based on these results, we selected the co-culture conditions of the effector to target ratio (5:1), BiTE concentration (100 ng/ml), and co-culture period (48 hours).

In the assay performed under these conditions, the cytotoxic activity of T cells in freshly isolated cells from lung tumor tissues was analyzed under a co-culture with a tumor cell line (U251) expressing EphA2 and EphA2-specific BiTE. In the immunologically “hot” and “cold” clusters, the cytotoxic activity of T cells in lung tumor tissue was greater for the “hot” cluster than the “cold” cluster (Fig. 1B). We also evaluated other factors related to the cytotoxic activity of T cells. Regarding the smoking status, the cytotoxic activity of T cells was stronger in smokers than in non-smokers (Fig. 1B).

**Figure 2:**
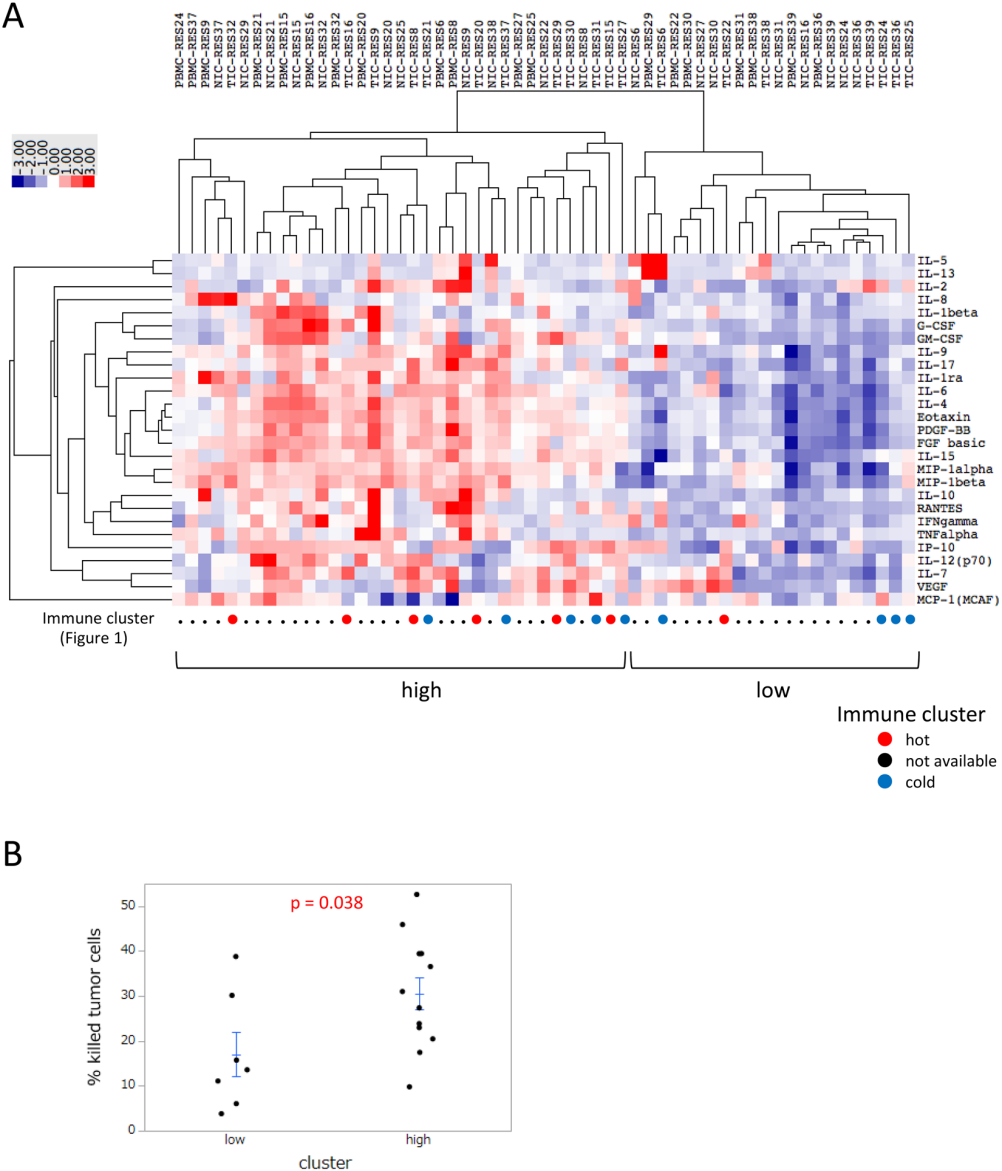
Cytokine production in NSCLC patients. (**A**) Cluster analysis for cytokine production in a co-culture with PBMC, normal lung-infiltrated cells (NIC), and lung tumor-infiltrated cells (TIC) (n = 19). The concentrations of 27 cytokines were measured in co-cultured supernatants. A hierarchical clustering algorithm was applied using the uncentered correlation coefficient as a measure of similarity and the method of average linkage by Cluster 3.0 and TreeView software. Individual data were transformed to Z scores for standardization purposes. (**B**) Comparison of the cytotoxic activity of T cells in the “high” and “low” cytokine clusters. The cytotoxic activity of T cells from tumor tissue in each cluster was evaluated (n = 19). Each dot represents one patient. Data were combined from triplicate experiments. The mean ± SEM was shown. An unpaired two-tailed Student’s *t*-test was used to examine the significance of differences between samples, with a p value < 0.05 indicating a significant difference.

In addition to the cytotoxic activity of T cells, we analyzed cytokine production under the same co-culture conditions. In this assay, we measured the cytokine concentrations of co-cultured supernatants not only for isolated cells from lung tumor tissues, but also normal lung tissues and PBMC. Based on these results, we performed a cluster analysis of multiple cytokines. The heat map of cytokines showed two clearly separated clusters, consisting of “high” and “low” cytokine concentrations (Fig. 2A). In these clusters, the cytotoxic activity of T cells in lung tumor tissue was higher for the “high” cytokine cluster than for the “low” cytokine cluster (Fig. 2B). Similar to immune profiles, cytokine production by PBMC, normal lung tissues, and tumor tissues partially correlated with each other (Supplementary Fig. S5).

### Analysis of peripheral blood

Based on the analysis of immune profiles and cytokine production from clinical samples of NSCLC patients, we hypothesized that there are some correlating factors among peripheral blood, normal lung tissues, and tumor tissues. Since the need to monitor T cell responses in the tumor microenvironment is increasing, we investigated relationships among various factors in peripheral blood and the cytotoxic activity of T cells in lung tumor tissues as a step towards the development of companion diagnostics using blood samples for cancer immunotherapy. Regarding immunological factors in peripheral blood, we focused on immune profiles, cytokine production, and the cytotoxic activity of T cells in peripheral blood.

**Figure 3:**
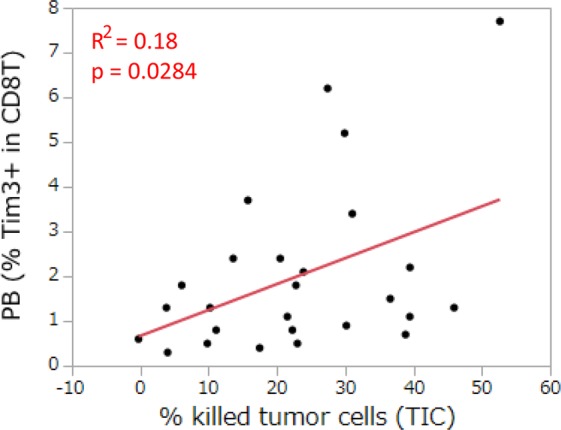
Relationship between the immune profile in peripheral blood and the cytotoxic activity of T cells in lung tumor tissues. The ratio of Tim-3^+^/CD8^+^ T cells in peripheral blood (PB) was analyzed for its relationship with the cytotoxic activity of T cells in lung tumor-infiltrated cells (TIC) (n = 26). Each dot represents one patient. Correlations between paired data were analyzed using Pearson’s correlation coefficient.

**Figure 4:**
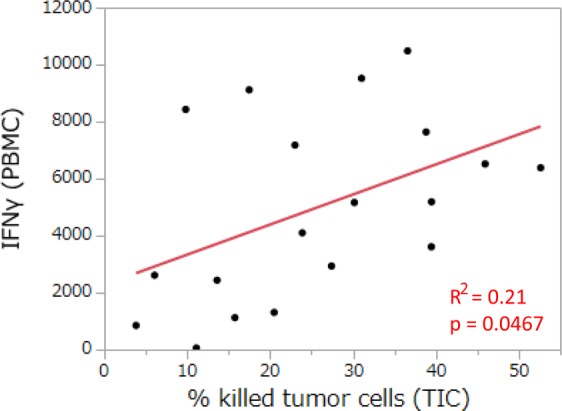
Relationship between cytokine production in PBMC and the cytotoxic activity of T cells in lung tumor tissues. The concentration of IFNγ in a co-culture with PBMC was analyzed for its relationship with the cytotoxic activity of T cells in lung tumor-infiltrated cells (TIC) (n = 19). Each dot represents one patient. Correlations between paired data were analyzed using Pearson’s correlation coefficient. The unit for the concentration of cytokines is pg/ml.

Concerning immune profiles, we analyzed 15 factors in T cells from peripheral blood. Among the 15 factors of the immune profile, only the ratio of Tim-3^+^/CD8^+^ T cells correlated with the cytotoxic activity of T cells in lung tumor tissues (Fig. 3, Supplementary Fig. S6). We then evaluated 27 cytokines in the co-culture of PMBC and the tumor cell line with BiTE. Among the 27 cytokines analyzed, only the concentration of IFNγ correlated with the cytotoxic activity of T cells in lung tumor tissue (Fig. 4, Supplementary Fig. S7).

**Figure 5:**
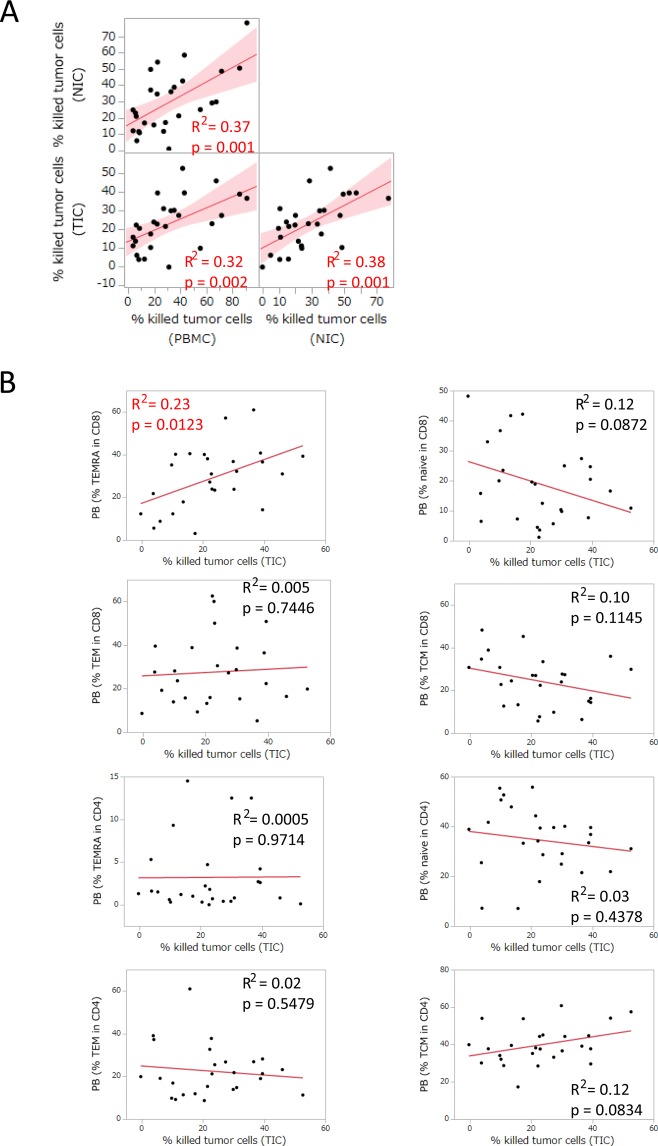
Relationships among the cytotoxic activity of T cells in peripheral blood, normal lung tissues, and tumor tissues. (**A**) The cytotoxic activity of T cells in PBMC was analyzed for its relationship with that in normal lung-infiltrated cells (NIC) and lung tumor-infiltrated cells (TIC) (n = 27). Each dot represents one patient. Data were combined from triplicate experiments. Correlations between paired data were analyzed using Pearson’s correlation coefficient. (**B**) The relationship between the phenotype of peripheral T cells and T cell cytotoxicity in lung tumor tissue. The ratio of naïve (CD27^+^ and CD45RA^+^), central memory (TCM, CD27^+^/CD45RA^−^), effector memory (TEM, CD27^−^/CD45RA^−^), and effector memory re-expresses CD45RA (TEMRA, CD45RA^+^/CD27^−^) in CD4^+^ and CD8^+^ T cells was analyzed for correlations with the cytotoxic activity of T cells in lung tumor tissue. Correlations between paired data were analyzed using Pearson’s correlation coefficient.

We then investigated relationships among the cytotoxic activity of T cells from peripheral blood, normal lung tissues, and tumor tissues. Measurements of the cytotoxicity of T cells in the co-culture of the tumor cell line with BiTE revealed correlations among the cytotoxic activity of T cells from peripheral blood, normal lung tissues, and tumor tissues (Fig. 5A). In the same co-culture experiments, the IFNγ secretion assay for CD4^+^ and CD8^+^ T cells showed some relationships among peripheral blood, normal lung tissues, and tumor tissues (Supplementary Fig. S8). We then evaluated the relationship between the phenotype of peripheral T cells and T cell cytotoxicity in lung tumor tissues. Among naïve, central memory (TCM), effector memory (TEM), and effector memory re-expresses CD45RA (TEMRA) T cells, the ratio of TEMRA in CD8^+^ T cells correlated with the cytotoxic activity of T cells in lung tumor tissue (Fig. 5B). TEMRA in CD8^+^ T cells also correlated with the cytotoxic activity of peripheral T cells (Supplementary Fig. S9).

We performed a multivariate analysis to detect predictive factors in peripheral blood for T cell cytotoxicity in lung tumor tissues. Among the six factors analyzed, the ratio of Tim-3^+^/CD8^+^ T cells and peripheral T cell cytotoxicity correlated with the cytotoxic activity of T cells in lung tumor tissues. Peripheral T cell cytotoxicity was found to be the most predictive factor (Supplementary Table S2).

### TCR repertoire analysis

In order to clarify the reasons for the strong correlations observed among the cytotoxic activity of T cells in peripheral blood, normal lung tissues, and lung tumor tissues, we analyzed their TCR repertoires of CD8^+^ T cells. In this analysis, we evaluated the TCR repertoires of six patients: two showed strong, two showed moderate, and two showed weak cytotoxicity (Table 1).

**Table 1:** Cytotoxicity of T cells (%) from peripheral blood, normal lung tissues, and tumor tissues for the TCR repertoire analysis.

	PBMC	NIC	TIC	Immune profiling
RES 4	34.3	36.1	29.9	hot
RES 8	18.8	37.2	17.5	hot
RES 19	18.8	49.8	10.2	cold
RES 23	14.0	17.0	4.0	hot
RES 24	9.7	11.7	3.8	cold
RES 38	86.9	50.5	38.8	N/A

**Figure 6:**
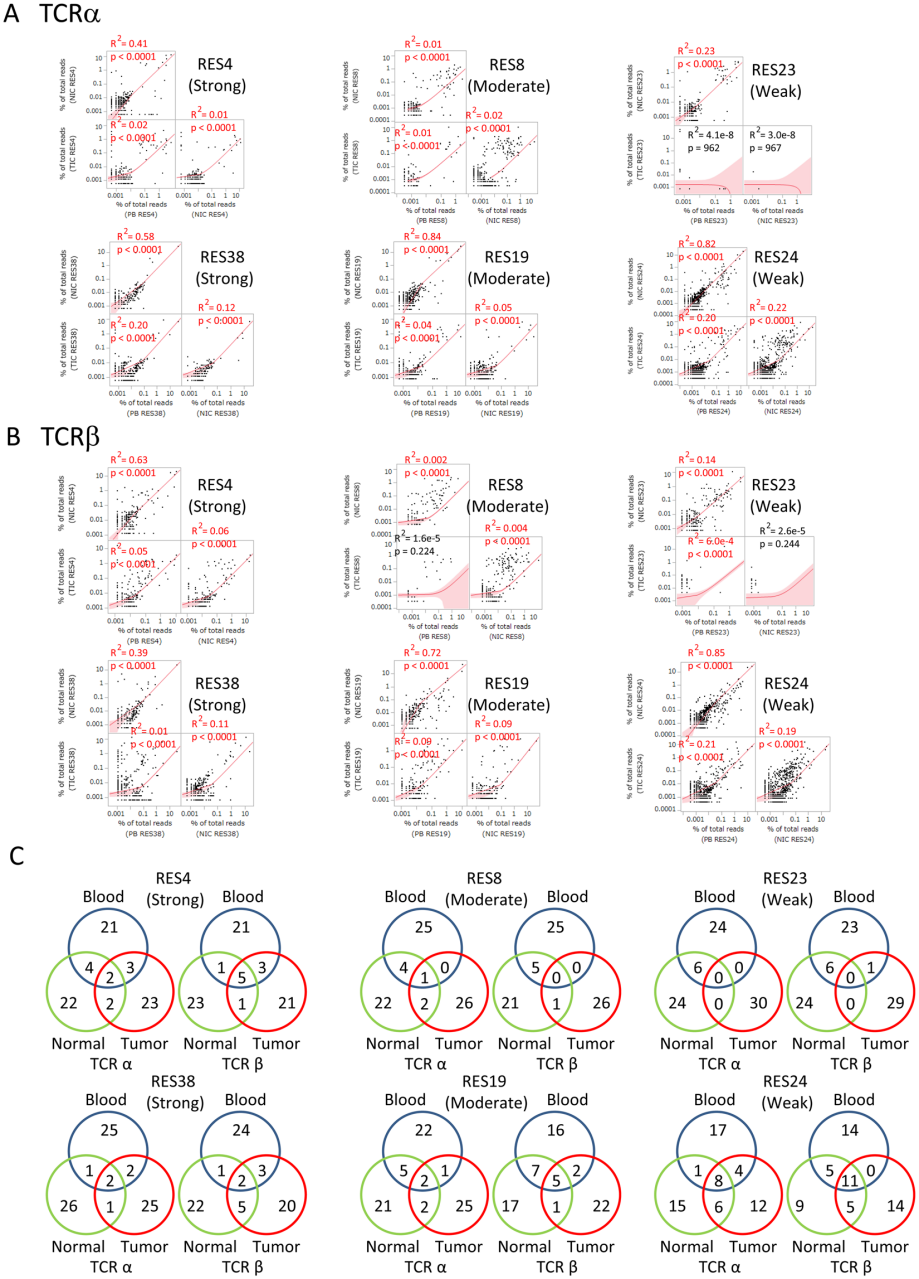
TCR repertoire analysis of CD8^+^ T cells from peripheral blood, normal lung tissues, and tumor tissues. The distribution of the TCRα (**A**) and TCRβ (**B**) clonotypes of CD8^+^ T cells was compared among peripheral blood, normal lung tissues, and tumor tissues in patients with strong (RES4 and RES38), moderate (RES8 and RES19), and weak (RES23 and RES24) cytotoxicities. Clonotypes with functional CDR3 were plotted. Correlations between paired data were analyzed using Pearson’s correlation coefficient. Frequencies were calculated based on total reads. (**C**) Comparisons of 30 of the most abundant TCRα and TCRβ clonotypes among peripheral blood, normal lung tissues, and tumor tissues.

The TCR repertoire analysis showed the enrichment of certain functional CDR3 sequences of TCRα and TCRβ among peripheral blood, normal lung tissues, and tumor tissues (Fig. 6A,B). Although a strong correlation was observed for the frequency of shared CDR3 sequences between peripheral blood and normal lung tissues, the ratios of most of their frequencies were less than 0.1%. On the other hand, the frequencies of many clonotypes in lung tumor tissues were higher than those in peripheral blood and normal lung tissues. A comparison of 30 of the most abundant TCR clonotypes among peripheral blood, normal lung tissues, and tumor tissues showed a certain number of shared TCR clonotypes (Fig. 6C). In contrast, the analysis of TCR clonotypes among the six patients indicated a weak correlation for peripheral blood, normal lung tissues, and tumor tissues (Supplementary Fig. S10A,B).

### Prediction of effects of nivolumab using peripheral blood

**Figure 7:**
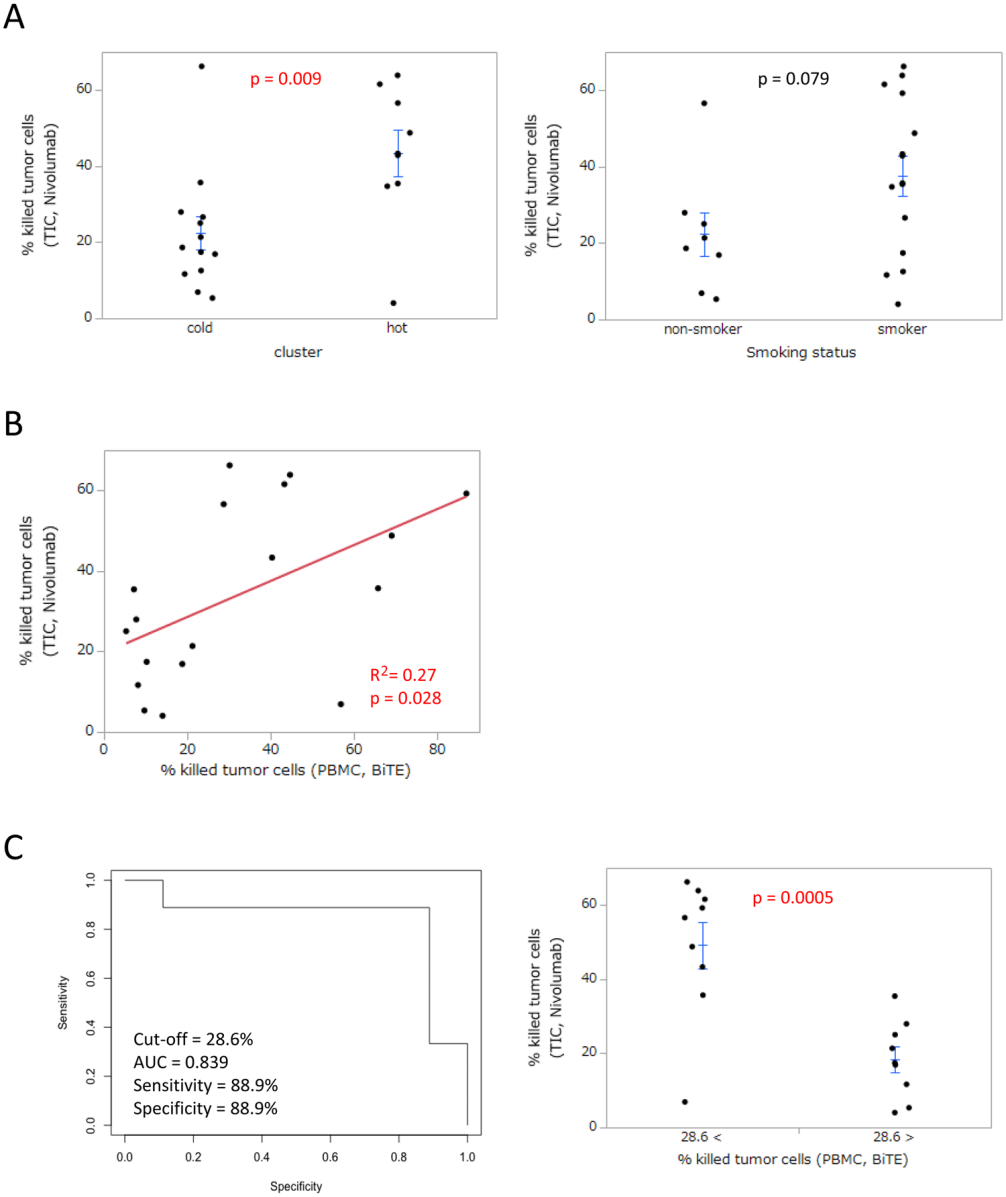
Evaluation of effects of nivolumab in lung tumor tissue. (**A**) Regarding immune clustering (n = 22) and the smoking status (n = 23), the effects of nivolumab on tumor-infiltrating T cells were evaluated. Each dot represents one patient. Data were combined from triplicate experiments. The mean ± SEM was shown. An unpaired two-tailed Student’s *t*-test was used to examine the significance of differences between samples, with a p value < 0.05 indicating a significant difference. (**B**) Relationship between peripheral T cell cytotoxicity and the effects of nivolumab in lung tumor tissues. The cytotoxic activity of T cells in PBMC was analyzed for its relationship with that enhanced by nivolumab in lung tumor-infiltrated cells (TIC) (n = 18). Each dot represents one patient. Data were combined from triplicate experiments. Relationships between paired data were analyzed using Pearson’s correlation coefficient. (**C**) Receiver operating characteristic (ROC) curves for peripheral T cell cytotoxicity for differentiating between “responders” (n = 9) and “non-responders” (n = 9) to the effects of nivolumab in lung tumor tissues (left). Comparison of the effects of nivolumab between patients with high (>28.6%, n = 9) and low (<28.6%, n = 9) peripheral T cell cytotoxicities (right). Each dot represents one patient. Data were combined from triplicate experiments. The mean ± SEM was shown. An unpaired two-tailed Student’s *t*-test was used to examine the significance of differences between samples, with a p value < 0.05 indicating a significant difference.

We evaluated the predictive power of peripheral T cell cytotoxicity for the effects of nivolumab in the tumor microenvironment. In this analysis, we also employed the assay system for the cytotoxic activity of T cells using BiTE. By using BiTE with nivolumab, the effects of nivolumab on T cell cytotoxicity in lung tumor tissues were evaluated. In the immunologically “hot” and “cold” clusters (Fig. 1A), the effects of nivolumab were greater in the “hot” cluster than in the “cold” cluster. Regarding the smoking status, the effects of nivolumab were slightly stronger in smokers (Fig. 7A). We analyzed PD-L1 expression on tumor cells by immunohistochemistry using anti-PD-L1 antibodies (clone 28-8 and clone SP142). We found that in contrast to <1% PD-L1 expression in patients with weak cytotoxicity (RES23), patients with strong (RES38) and moderate (RES19) cytotoxicities showed 1–49% PD-L1 expression on tumor cells (Table 1, Supplementary Fig. S11).

Based on these results, we evaluated the predictive power of peripheral T cell cytotoxicity for the effects of nivolumab in lung tumor tissue. In the analysis of peripheral blood and lung tumor tissues from NSCLC patients, the cytotoxic activity of T cells from peripheral blood correlated with the effects of nivolumab on the cytotoxicity of T cells in lung tumor tissues (Fig. 7B). In order to assess the predictive power of peripheral T cell cytotoxicity, we analyzed the area under the receiver operating characteristic (ROC) curve (AUC). The AUC was 0.839 for differentiating “responders” (n = 9) from “non-responders” (n = 9) to the effects of nivolumab in lung tumor tissues with a cut-off value of 28.6% (sensitivity = 88.9%, specificity = 88.9%) (Fig. 7C). In the preliminary analysis of peripheral T cell cytotoxicity in PD-1 inhibitor recipients, we found that patients with strong cytotoxicity (n = 3) had a higher rate of treatment continuation than those with weak cytotoxicity (n = 3) (Supplementary Fig. S12). These results imply that the analysis of peripheral T cell cytotoxicity predicts the effects of nivolumab in the tumor microenvironment.

## Discussion

In the present study, we demonstrated that the cytotoxic activity of tumor-infiltrated T cells closely correlated with that of peripheral T cells using BiTE technology. Furthermore, we found that the cytotoxicity of peripheral T cells has potential as a predictor of the effects of nivolumab in the tumor microenvironment. These results suggest that the monitoring of peripheral T cell activity predicts the effects of immune responses at tumor sites in cancer patients. Our TCR repertoire analysis showed a certain number of shared TCR clonotypes in peripheral blood, normal lung, and tumor-infiltrated CD8^+^ T cells, and the frequencies of many clonotypes in lung tumor tissues were higher than those in peripheral blood and normal lung tissues. These results imply that a certain number of antigen-specific T cells at tumor sites appear in normal lung tissues and peripheral blood. This is consistent with previous findings showing that tumor antigen-specific T cells may be detected within peripheral blood in cancer patients^10–12^. We speculate that the cytotoxic activity of tumor antigen-specific T cells derived from the tumor microenvironment may be detected in peripheral blood using our analysis.

In a phase 1 study of CD19-specific BiTE (blinatumomab) in non-Hodgkin lymphoma patients, an analysis of T cell subpopulations indicated that the expansion of T cells induced by blinatumomab was primarily due to the subset of CD4^+^ and CD8^+^ effector T cells, and not to naïve or central memory T cells^13^. The lack of a T-cell co-stimulation of BiTE via CD28 will prevent the differentiation of naïve T-cell clones, and explains the predominant engagement for the redirected lysis of effector T cells, which are no longer in need of a CD28 co-stimulation. In our analysis, the ratio of TEMRA in CD8^+^ T cells correlated with the cytotoxic activity of T cells in peripheral blood and lung tumor tissues. Therefore, in our T-cell functional analysis using BiTE technology, effector T cells are estimated to exhibit predominant cytotoxic activity against tumor cells.

The identification of the total number, type, and activity of tumor antigen-specific T cells in the tumor microenvironment is one of the main focuses in cancer immunology and immunotherapy. However, difficulties are associated with evaluating the cytotoxic activity of tumor-infiltrated T cells in cancer patients. In our analysis with BiTE technology, we showed that the cytotoxicity of tumor-infiltrated T cells was related to the immune profiling of tumor tissue and smoking status of NSCLC patients. Our results are partially consistent with a previous study on the cytometric profiling of lung cancer, which identified an immunologically “hot” cluster with abundant CD8^+^ T cells expressing high levels of PD-1 and TIM-3. The “hot” cluster was highly enriched for the expression of genes associated with T cell cytotoxic function and strong PD-L1 expression by IHC, which has been developed for companion diagnostics to anti-PD-1 immunotherapy for NSCLC^14,15^. Regarding the smoking status, clinical trials have shown that the therapeutic effects of the anti-PD-1 blocking antibody were better for smoking patients than for non-smoking patients with NSCLC^16^. One potential explanation for the lower efficacy in non-smokers is the lower tumor mutational load and associated neoantigens in these patients, which leads to weaker immunogenicity^17^. Therefore, our results for the cytotoxicity of tumor-infiltrated T cells have some correlation with these findings on immune profiling and the smoking status of NSCLC patients. In our analysis, each treated well consisted of U251 cells and freshly isolated cells from tumor tissues with EphA2/CD3 BiTE. These freshly isolated cells from tumor tissues consisted not only of T cells, but also other types of cells, including macrophages. Therefore, the cytotoxicity response in our assay appears to partially reflect that in the tumor microenvironment.

There are several limitations in the present study. Due to technical difficulties, we used tumor cell lines, not freshly isolated autologous tumors to assess the cytotoxicity of T cells. Therefore, we mainly evaluated the activity of peripheral or tumor-infiltrated T cells themselves, except for the characteristics of autologous tumors in NSCLC patients. In contrast, PD-L1 expression for companion diagnostics to anti-PD-1 immunotherapy is analyzed on tumor cells, not immune cells. PD-L1 expression on tumor cells is estimated to reflect the interaction between tumor and immune cells in the tumor microenvironment, partially due to the induction of PD-L1 expression by IFNγ from activated T cells^18^. Furthermore, we analyzed patients with early-stage disease in the present study. Without surgically resected specimens of early-stage disease, there was an insufficient number of tumor-infiltrated T cells to assess T cell cytotoxicity for biopsy specimens from patients with late-stage disease. Therefore, we are now conducting a clinical study to analyze peripheral T cell cytotoxicity in PD-1 inhibitor recipients with late-stage disease in order to link assay results to clinical outcomes.

In conclusion, by using a simple method with BiTE technology, we herein demonstrated that peripheral T cell activity closely correlated with T cell function in the tumor microenvironment. These results imply further applications to blood-based immune monitoring systems and predictive biomarkers for cancer immunotherapy.

## Methods

### Sample preparation

NSCLC tumor tissue, matched normal lung tissue, and peripheral blood were obtained from 39 patients between May 2015 and October 2016.

White blood cells were extracted from peripheral blood by lysis with BD Pharm lyse (BD Biosciences) red blood cell lysis buffer and then subjected to a FACS analysis. PBMC were isolated from peripheral blood by gradient density centrifugation using Lymphoprep (Axis Shield), and this was followed by a T-cell functional analysis.

Fresh tumor or normal lung tissues were minced in a 6-cm dish and digested to a single cell suspension using a Tumor Dissociation Kit for humans (Miltenyi Biotec) and gentleMACS Dissociator (Miltenyi Biotec) according to the manufacturer’s instructions. The cell suspension was applied to a 70-μm nylon cell strainer (BD Biosciences) with the lysis of red blood cells by BD Pharm lyse. Dead cells and debris were removed by centrifugation in isodensity Percoll solution (Pharmacia Biotech), followed by FACS and T-cell functional analyses. The remaining cells were cryopreserved in liquid nitrogen for the TCR repertoire analysis.

### Flow cytometric analysis

Surface marker staining was performed after the FcR block using Human TruStain FcX Fc Receptor blocking solution (BioLegend). Cells were incubated with the Live/Dead Fixable Yellow Dead Cell Stain Kit (Life Technologies). Surface marker-stained cells were analyzed on BD LSRFortessa with FACSDiva software (BD Biosciences). The gating strategy of the FACS analysis was shown in Supplemental Fig. 13. The following antibodies were used for FACS staining: anti-CD45RA-FITC (clone HI100), anti-CD25-PE (BC96), anti-4-1BB-BV421 (4B4-1), anti-CD8-BV510 (RPA-T8), anti-CD103-BV605 (Ber-ACT8), anti-CD4-BV711 (OKT4), anti-Tim-3-APC (F38-2E2), anti-CD3-Alexa Fluor 700 (UCHT1), anti-CD15-BV510 (W6D3), anti-CD11b-BV605 (M1/70), anti-CD45-BV786 (HI30), anti-CD14- Alexa Fluor 700 (HCD14), and IgG1 isotype control (MOPC-21) purchased from Biolegend. Anti-ICOS-PerCP eFluor 710 (ISA-3) and IgG1 isotype control (P3.6.2.8.1) were purchased from eBioscience. Anti-OX-40-PE-CF594 (ACT35), anti-PD-1-PE Cy7 (EH12.1), and anti-CCR3-PE-CF594 (5E8) were purchased from BD Bioscience.

### Construction of the EphA2-specific T-cell engager

The construction of the EphA2-specific engager containing EphA2-specific scFv 4H5^19^, a short serine-glycine linker, and CD3-specific scFv derived from OKT3 is described elsewhere^20,21^. Specifically, the EphA2-specific engager consists of the 4H5 heavy-chain, a glycine (G) serine (S) linker [(G4S)3], the 4H5 light-chain^19^, a short G4S linker, the OKT3 heavy-chain, a (G4S)3 linker, and the OKT3 light-chain. A 6×His-Myc tag was inserted at the C terminus before the stop codon (Supplementary Table S3). This recombinant protein was custom-made by Thermo Fisher Scientific. Briefly, EphA2-specific engager DNA was synthesized and subcloned into the pcDNA3.3 vector. This plasmid vector was transfected into Expi293™ cells. After the culturing of cells, the protein was purified by His-tag affinity chromatography from the culture supernatant.

### Functional analysis of T cells

The U251 cell line was kindly provided by Dr. Yasuko Mori (Kobe University, Japan). Cell line authentication by short tandem repeat (STR) profiling and mycoplasma testing were performed in the JCRB Cell Bank. U251 cells were plated on 96-well flat-bottomed cell culture plates (Corning) at a density of 1 × 10^4^ cells per well with PRMI medium 1640 (Nacalai Tesque) containing 10% fetal bovine serum (FBS; HyClone, Thermo Scientific). After 24 hours, 5 × 10^4^ PBMC or freshly isolated cells from normal lung or tumor tissues were added to plates with 100 ng/ml of EphA2/CD3 BiTE. After a 48-hour co-culture, culture supernatants were cryopreserved, non-adherent cells were removed by gentle washing four times with RPMI medium 1640 containing 10% FBS, and the remaining adherent viable cells were detected by the 3-(4,5-dimethylthiazol-2-yl)-5-(3-carboxymethoxyphenyl)-2-(4-sulfophenyl)-2H-tetrazolium (MTS) assay (CellTiter 96 aqueous one solution cell proliferation assay; Promega). The assay was performed in triplicate. The calculation of EphA2/CD3 BiTE-mediated killing was based on the degree of the reduction in viable target cells with the following formula:

$$ \% \,{\rm{EphA}}2/{\rm{CD}}3\,\mathrm{BiTE} \mbox{-} \mathrm{mediated}\,{\rm{killing}}=[1-({\rm{absorbance}}\,{\rm{of}}\,{\rm{treated}}\,{\rm{wells}})/({\rm{absorbance}}\,{\rm{of}}\,\mathrm{non} \mbox{-} \mathrm{treated}\,{\rm{wells}})]\times 100$$

Each treated well consisted of 1 × 10^4^ U251 cells and 5 × 10^4^ PBMC or freshly isolated cells from normal lung or tumor tissues with 100 ng/ml of EphA2/CD3 BiTE. Each non-treated well consisted of 1 × 10^4^ U251 cells and 5 × 10^4^ PBMC or freshly isolated cells from normal lung or tumor tissues without EphA2/CD3 BiTE.

Collected non-adherent cells were analyzed by the IFNγ secretion assay (Miltenyi Biotec) according to the manufacturer’s instructions. The gating strategy for the IFNγ secretion assay was shown in Supplemental Fig. 14. Cryopreserved culture supernatants were analyzed by the Bio-Plex Pro™ Human Cytokine 27-plex Assay (Bio-Rad).

In the evaluation of T cell cytotoxicity enhanced by nivolumab, 1 μg/ml of nivolumab (provided by Ono Pharmaceutical) was added to plates with 100 ng/ml of EphA2/CD3 BiTE. As a control, 1 μg/ml of human IgG4 (Abcam) was added to plates with 100 ng/ml of EphA2/CD3 BiTE. The calculation of % cytotoxicity was based on the degree of the reduction induced in viable target cells by nivolumab using the following formula:

$$\begin{array}{ccl}{\rm{Percentage}}\,{\rm{cytotoxicity}} & = & [1-({\rm{absorbance}}\,{\rm{of}}\,{\rm{treated}}\,{\rm{wells}}\,{\rm{with}}\,{\rm{nivolumab}})\\  &  & /({\rm{absorbance}}\,{\rm{of}}\,{\rm{treated}}\,{\rm{wells}}\,{\rm{with}}\,{\rm{control}}\,{\rm{IgG}}4)]\times 100\end{array}$$

### TCR repertoire analysis

Cryopreserved PBMC or isolated cells from normal lung or tumor tissues were thawed and sorted into CD8^+^ T cells with the FACSAria II cell sorter (Becton Dickinson). Total RNA was extracted and purified with the RNeasy Mini Kit (Qiagen) according to the manufacturer’s instructions. RNA amounts and purity were measured with Agilent 2200 TapeStation (Agilent Technologies). A next-generation sequencing analysis was performed with unbiased TCR repertoire analysis technology developed by Repertoire Genesis Inc.. Unbiased adaptor-ligation PCR was performed according to a previous study^22^. Total RNA was converted to complementary DNA (cDNA) with Superscript III reverse transcriptase (Invitrogen). A BSL-18E primer containing polyT18 (Supplementary Table S4) and a NotI site was used for cDNA synthesis. After cDNA synthesis, double-strand (ds)-cDNA was synthesized with *E*. *coli* DNA polymerase I (Invitrogen), *E*. *coli* DNA Ligase (Invitrogen), and RNase H (Invitrogen). ds-cDNAs were blunted with T4 DNA polymerase (Invitrogen). A P10EA/P20EA adaptor was ligated to the 5′ end of ds-cDNA and then cut with the NotI restriction enzyme. After removal of the adaptor and primer with the MinElute Reaction Cleanup kit (Qiagen), PCR was performed with KAPA HiFi DNA Polymerase (Kapa Biosystems) using TCR α-chain constant region-specific (CA1) or TCR β-chain constant region-specific primers (CB1) and P20EA (Supplementary Table S4). PCR conditions were as follows: 98 °C (20 sec), 65 °C (30 sec), and 72 °C (1 min) for 20 cycles. Second PCR was performed with CA2 or CB2 and P20EA primers using the same PCR conditions. Amplicons were prepared by the amplification of second PCR products using P22EA-ST1 and CA-ST1-R or CB-ST1-R (Supplementary Table S4). After PCR amplification, index (barcode) sequences were added by amplification with Nextera XT index kit v2 setA (Illumina). The indexed amplicon products were mixed in an equal molar concentration and quantified by a Qubit 2.0 Fluorometer (Thermo Fisher Scientific). Sequencing was performed with the Illumina Miseq paired-end platform (2 × 300 bp).

All paired-end reads were classified by index sequences. The assignment of sequences was performed by identifying sequences with the highest identity in a data set of reference sequences from the international ImMunoGeneTics information system® (IMGT) database (http://www.imgt.org). Data processing, assignment, and aggregation were automatically performed using repertoire analysis software originally developed by Repertoire Genesis Inc. The nucleotide sequences of the CDR3 regions ranged from a conserved cysteine at position 104 (Cys104) of the IMGT nomenclature to a conserved phenylalanine at position 118 (Phe118), and the subsequent glycine (Gly119) was translated to deduced amino acid sequences. The copy numbers of identical unique sequence reads were automatically counted by RG software in each sample and then ranked in order of the copy number (Supplementary Table S5). The percentage occurrence frequencies of sequence reads with the TRAV, TRAJ, TRBV, and TRBJ genes in total sequence reads were calculated. Out of frame sequences were excluded from analyses. This appears to be essentially the same type of approach described elsewhere^23^.

### Immunohistochemistry

The rabbit monoclonal anti-human PD-L1 antibodies used were clone 28-8 (Abcam) and clone SP142 (Spring Bioscience). Heat-induced epitope retrieval with High pH Target Retrieval Solution (DAKO) was performed. Endogenous peroxidase activity was blocked by incubating in 0.3% hydrogen peroxidase and 0.1% sodium azide contained 0.01 M phosphate-buffered saline, and the EnVision plus system (DAKO) was used for secondary detection. The final product was visualized by 3-3′ diaminobenzidine.

### Statistical analysis

An unpaired two-tailed Student’s *t*-test was used to examine the significance of differences between samples, with a p value < 0.05 indicating a significant difference. Relationships between paired data were analyzed using Pearson’s correlation coefficient by JMP software (SAS Institute, Inc.). A hierarchical clustering algorithm was applied using the uncentered correlation coefficient as a measure of similarity and the method of average linkage by Cluster 3.0 and TreeView software. Individual data were transformed to Z scores for standardization purposes. In the drawing of receiver operating characteristic (ROC) curves and estimation of the area under the ROC curve (AUC), R software was used to quantify the ability to differentiate between “responders” (n = 9) and “non-responders” (n = 9) to the effects of nivolumab in lung tumor tissues. We applied a multivariate linear regression analysis to detect variables associated with T cell cytotoxicity in lung tumor tissues. In this analysis, the following were included as independent (predictor) variables: the ratio of Tim-3^+^/CD8^+^ T cells, the ratio of TEMRA/CD8^+^ T cells, and peripheral T cell cytotoxicity were included as continuous variables, while smoking status, histology, and stage were added to the analysis. The values of Tim-3^+^/CD8^+^ T cells, TEMRA/CD8^+^ T cells, and peripheral T cell cytotoxicity were transformed by a logarithmic transformation. The transformed values of these three values were then standardized. Multiple imputation was used to account for two missing datasets (Tim-3^+^/CD8^+^ and TEMRA/CD8^+^) with the MICE package of R Statistical Software. R^2^ indicates the proportion of variability in the observed responses that may be attributed to changes in predictor variables. A p value < 0.05 was considered to be significant.

### Study approval

The present study was conducted according to the principles of the Declaration of Helsinki. The study protocol was approved by the Osaka University Hospital Ethics Committee, and written informed consent was obtained from participants prior to their inclusion in the study.

## Supplementary information

Supplementary Information

## Data Availability

The authors declare that the main data supporting the results of the present study are available within the article and its Supplementary Information files. Extra data are available from the corresponding author upon request.
