# Supplementary material for: Peripheral T cell cytotoxicity predicts T cell function in the tumor microenvironment
Source: Sci Rep. 2019 Feb 22;9:2636. doi: 10.1038/s41598-019-39345-5 (PMC6385254; doi:10.1038/s41598-019-39345-5)
Supplement: Supplementary file 1 — Supplementary Information [file 41598_2019_39345_MOESM1_ESM.pdf]

Peripheral T cell cytotoxicity predicts T cell function in the tumor microenvironment

Kota Iwahori[1,2]*, Yasushi Shintani[3], Soichiro Funaki[3], Yoko Yamamoto[1,3], Mitsunobu Matsumoto[1,4], Tetsuya Yoshida[4,5], Akiko Morimoto-Okazawa[1], Atsunari Kawashima[1], Eiichi Sato[6], Stephen Gottschalk[7], Meinoshin Okumura[3], Atsushi Kumanogoh[2], Hisashi Wada[1]

1 Department of Clinical Research in Tumor Immunology, Graduate School of Medicine, Osaka University, Suita, Osaka, Japan.
2 Department of Respiratory Medicine and Clinical Immunology, Graduate School of Medicine, Osaka University, Suita, Osaka, Japan.
3 Department of General Thoracic Surgery, Graduate School of Medicine, Osaka University, Suita, Osaka, Japan.
4 Shionogi & Co., Ltd., Osaka, Japan.
5 Department of Frontier Research in Tumor Immunology, Graduate School of Medicine, Osaka University, Suita, Osaka, Japan.
6 Department of Pathology (Medical Research Center), Institute of Medical Science, Tokyo Medical University, Tokyo, Japan.
7 Department of Bone Marrow Transplant and Cellular Therapy, St. Jude Children's Research Hospital, Memphis, TN, USA.

*To whom correspondence should be addressed:
Kota Iwahori
2-2 Yamadaoka, Suita, Osaka 565-0871, Japan.
TEL.: +81-6-6210-8413
E-mail address: iwahori@climm.med.osaka-u.ac.jp

Supplementary Table S1 Patient characteristics

All NSCLC cases		39
Age		
	Range	51-82
	Median	70
Sex		
	Male	25
	Female	14
Histology		
	Adenocarcinoma	33
	Squamous	4
	Adenosquamous	2
Stage		
	IA	14
	IB	16
	IIA	6
	IIB	1
	IIIA	1
	IIIB	1
Smoking status		
	Current smoker	7
	Former smoker	17
	Non-smoker	15

Supplementary Table S2

Multivariate analysis model of factors associated with T cell cytotoxicity in lung tumor tissues.

Variable	estimate	95% CI low		95% CI high	P value
Intercept	-0.285	-0.788	-	0.217	0.248
Smoking status	0.667	-0.014	-	1.347	0.054
Smoker					
Non-smoker					
Stage	-0.310	-1.019	-	0.400	0.371
I					
II and III					
Histology	-0.255	-1.119	-	0.609	0.543
Adenocarcinoma					
Non-adenocarcinoma					
% TEMRA in CD8+ T cells	0.197	-0.149	-	0.542	0.247
% Tim-3 in CD8+ T cells	0.390	0.041	-	0.739	0.031 *
Peripheral T cell cytotoxicity (%)	0.421	0.085	-	0.757	0.017 *

$R^2 = 0.663$

CI confidence interval

A p value < 0.05 (*) was considered to be significant.

Supplementary Table S3 Nucleotide and amino acid sequences of the EphA2-specific T-cell engager

Nucleotide sequence

1 atggactgga tctggcggat tctgttcctc gtgggagccg ccacaggcgc tcactcacag
61 gtgcagctgc tggaatctgg cggcggactg gtgcagcctg gcggcagcct gagactgagc
121 tgcgccgcca gcggcttcac cttcagcagc tacaccatga gctgggtccg gcaggctcct
181 ggacaggccc tggaatggat gggcaccatc agcagcggcg gcacctacac ctactacccc
241 gacagcgtga agggccggtt caccatcagc cgggacaacg ccaagaacag cctgtacctg
301 cagatgaaca gcctgagagc cgaggacaca gccgtgtact actgcgccag agaggccatc
361 ttcacctact ggggcagagg caccctggtc acaagcagcg gaggcggagg aagtggaggg
421 ggaggatcag gcggcggagg cagcgatatc cagctgaccc agagccctag cagcctgagc
481 gccagcgtgg gcgacagagt gaccatcaca tgcaaggcca gccaggacat caacaactac
541 ctgagctggt atcagcagaa gcccggccag gcccccagac tgctgatcta ccgggccaac
601 agactggtgg acggcgtgcc cgatagattc agcggcagcg gctacggcac cgacttcacc
661 ctgaccatca acaacatcga gtccgaggac gccgcctact acttctgcct gaagtacgac
721 gtgttcccct acaccttcgg ccagggcacc aaggtggaga tcaagtccgg aggtggtgga
781 tccgatatca aactgcagca gtcaggggct gaactggcaa gacctggggc ctcagtgaag
841 atgtcctgca agacttctgg ctacaccttt actaggtaca cgatgcactg ggtaaaacag
901 aggcctggac agggtctgga atggattgga tacattaatc ctagccgtgg ttatactaat
961 tacaatcaga agttcaagga caaggccaca ttgactacag acaaatcctc cagcacagcc
1021 tacatgcaac tgagcagcct gacatctgag gactctgcag tctattactg tgcaagatat
1081 tatgatgatc attactgcct tgactactgg ggccaaggca ccactctcac agtctcctca
1141 ggtggtggtg gttctggcgg cggcggctcc ggtggtggtg gttctgacat tcagctgacc
1201 cagtctccag caatcatgtc tgcatctcca ggggagaagg tcaccatgac ctgcagagcc
1261 agttcaagtg taagttacat gaactggtac cagcagaagt caggcacctc ccccaaaaga
1321 tggatttatg acacatccaa agtggcttct ggagtccctt atcgcttcag tggcagtggg
1381 tctgggacct catactctct cacaatcagc agcatggagg ctgaagatgc tgccacttat
1441 tactgccaac agtggagtag taacccgctc acgttcggtg ctgggaccaa gctggagctg
1501 aaatccggac cgcatcatca ccaccaccat atggaacaaa aacttatttc tgaagaagat
1561 ctgtaa

Amino acid sequence

1 mdwiwrilfl vgaatgahsq vqllesgggl vqpggslrls caasgftfss ytmswvrqap
61 gqalewmgti ssggtytyyp dsvkgrftis rdnaknslyl qmnslraedt avyycareai
121 ftywgrgtlv tssggggsgg ggsggggsdi qltqspssls asvgdrvtit ckasqdinny
181 lswyqqkpgq aprlliyran rlvdgvpdrf sgsgygtdft ltinniesed aayyfclkyd
241 vfpytfgqgt kveiksgggg sdiklqqsga elarpgasvk mscktsgytf trytmhwvkq
301 rpgqglewig yinpsrgytn ynqkfkdkat lttdksssta ymqlssltse dsavyycary
361 yddhycldyw gqgttltvss ggggsggggs ggggsdiqlt qspaimsasp gekvtmtcra
421 sssvsymnwy qqksgtspkr wiydtskvas gvpyrfsgsg Sgtsysltis smeaedaaty
481 ycqqwssnpl tfgagtklel ksgphhhhhh meqkliseed l

Supplementary Table S4 PCR primers used for adaptor ligation PCR

Primer	Sequence
BSL-18E	AAAGCGGCCGCATGCTTTTTTTTTTTTTTTTTTVN
P20EA	TAATACGACTCCGAATTCCC
P10EA	GGGAATTCGG
CA1	TGTTGAAGGCGTTTGCACATGCA
CA2	GTGCATAGACCTCATGTCTAGCA
CB1	GAACTGGACTTGACAGCGGAACT
CB2	AGGCAGTATCTGGAGTCATTGAG
P22EA-ST1-R	GTCTCGTGGGCTCGGAGATGTGTATAAGAGACAGCTAA TACGACTCCGAATTCCC
CA-ST1-R	TCGTCGGCAGCGTCAGATGTGTATAAGAGACAGGAGG GTCAGGGTTCTGGA
CB-ST1-R	TCGTCGGCAGCGTCAGATGTGTATAAGAGACAGGCTC AAACACAGCGACCTC

Supplementary Table S5 Number of sequence reads for the TCR repertoire analysis

Patient No.	Source	Total Reads	Assigned Reads	In-frame Reads	Unique Reads
(TCRa)					
RES4	PBMC	256242	215120	187030	10827
RES4	Normal	201955	184582	160955	6010
RES4	Tumor	212435	191425	164001	8566
RES8	PBMC	375264	308934	277039	13225
RES8	Normal	395027	343471	325941	16706
RES8	Tumor	317584	296397	268659	9419
RES19	PBMC	223493	198271	175655	10009
RES19	Normal	236582	202296	184505	3364
RES19	Tumor	155372	140527	124337	5833
RES23	PBMC	241417	206030	167911	12794
RES23	Normal	225951	192502	168241	6251
RES23	Tumor	132413	127607	126859	2828
RES24	PBMC	441229	383625	348484	12300
RES24	Normal	425158	367979	316495	13684
RES24	Tumor	411104	343913	302647	13076
RES38	PBMC	205070	175503	156854	5588
RES38	Normal	181834	167905	147023	2857
RES38	Tumor	196387	166511	156147	6417
(TCRb)					
RES4	PBMC	79894	62646	60600	5874
RES4	Normal	97520	78827	75280	4497
RES4	Tumor	111238	80474	76668	5723
RES8	PBMC	254014	181698	179198	8780
RES8	Normal	193245	132164	129855	6496
RES8	Tumor	379213	272536	269843	8958
RES19	PBMC	120111	81283	78493	6909
RES19	Normal	194680	152650	151602	2615
RES19	Tumor	96165	77658	75941	5243
RES23	PBMC	145844	98431	94927	9841
RES23	Normal	198616	143056	138308	5236
RES23	Tumor	43695	39723	21028	1771
RES24	PBMC	313661	247853	233845	8763
RES24	Normal	327340	254091	234074	9293
RES24	Tumor	311047	234541	228528	10061
RES38	PBMC	107423	77851	76085	5391
RES38	Normal	176451	150859	147843	3607
RES38	Tumor	210927	150387	145973	9593

Supplementary Table S6
Tumor proportion score (TPS)

Patient No.	TPS clone 28-8	TPS clone SP142	Peripheral cytotoxicity	Effects of nivolumab on tumors
RES16	< 1%	< 1%	28.7%	56.6%
RES17	< 1%	< 1%	40.3%	43.3%
RES19	1 - 49%	1 - 49%	18.8%	16.9%
RES20	< 1%	< 1%	69.0%	48.8%
RES23	< 1%	< 1%	14.0%	4.0%
RES25	< 1%	< 1%	8.2%	11.7%
RES30	< 1%	< 1%	10.2%	17.4%
RES33	< 1%	< 1%	7.7%	28.0%
RES37	< 1%	< 1%	65.7%	35.7%
RES38	< 1%	1 - 49%	86.9%	59.3%

Supplementary Figure S1

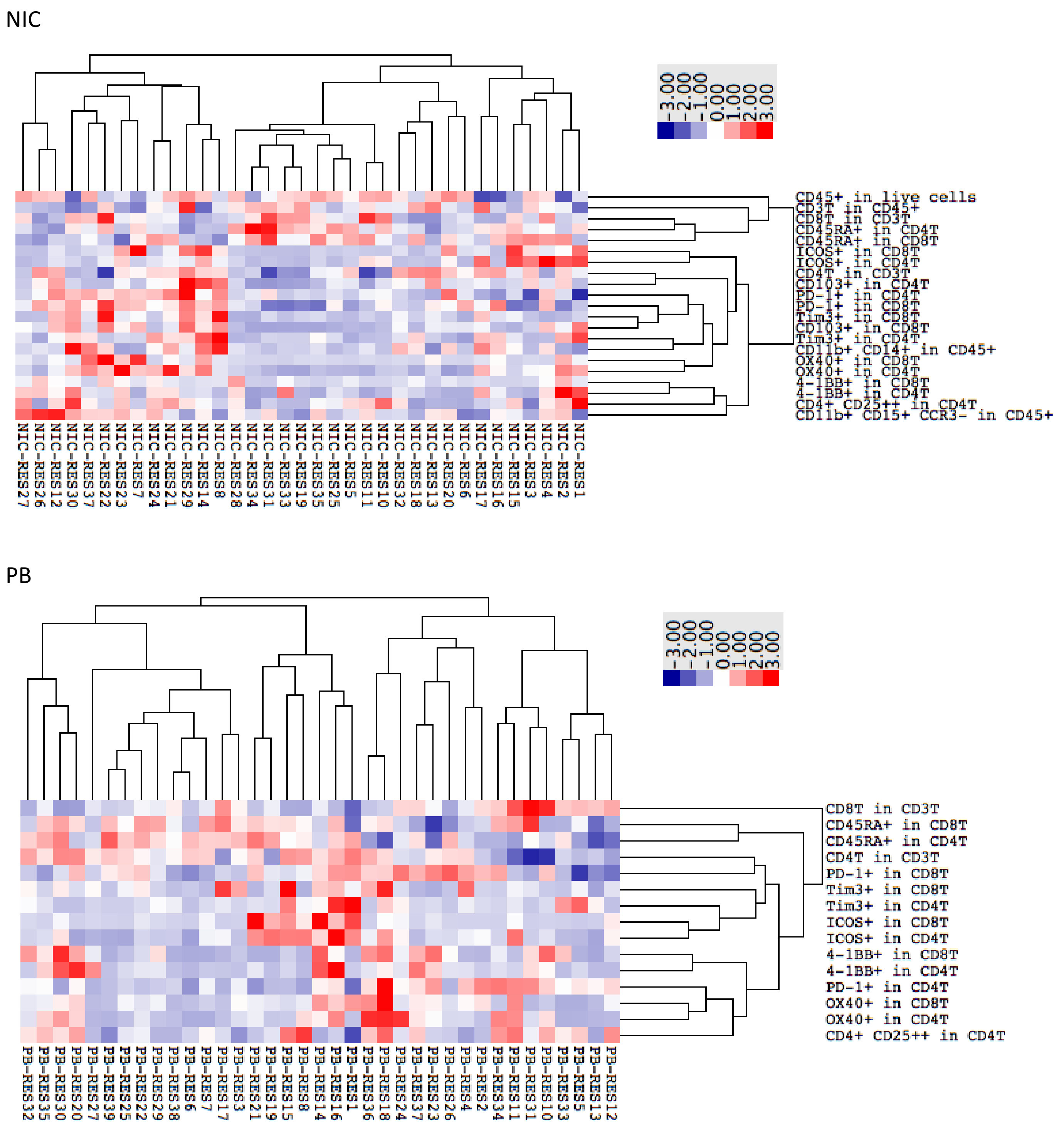

Supplementary Figure S1. Immune profiling of NSCLC patients. Cluster analysis for the immune profiling of peripheral blood (PB) (n = 37) and normal lung-infiltrating cells (NIC) (n = 35) from NSCLC patients.

Supplementary Figure S2

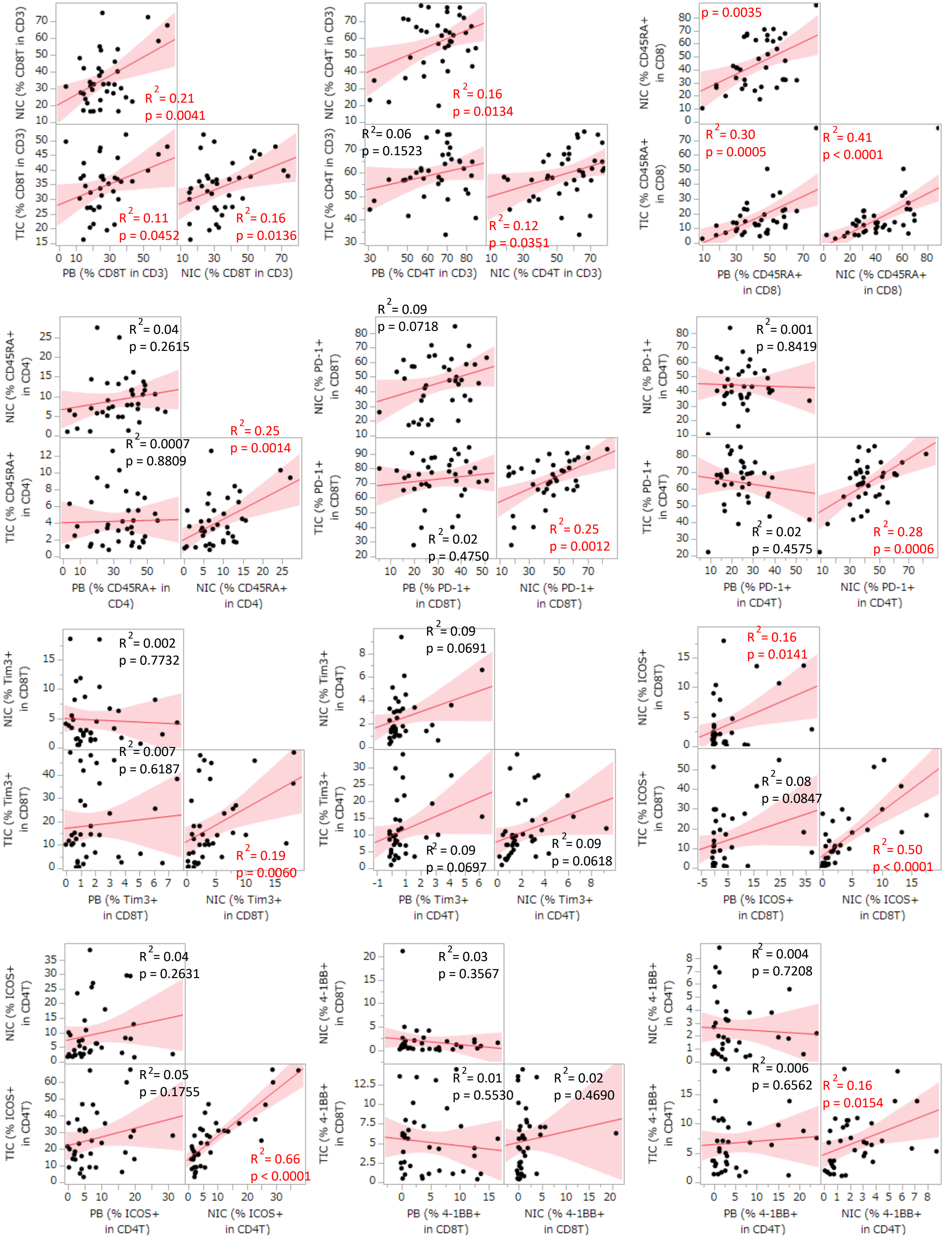

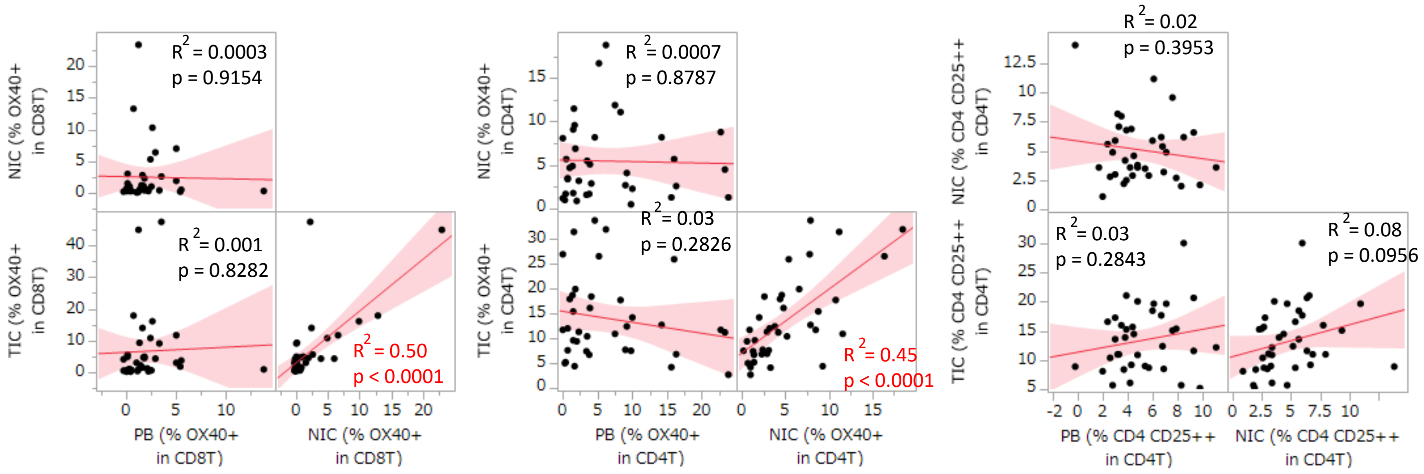

Supplementary Figure S2. Immune profile relationships among peripheral blood, normal lung tissues, and tumor tissues. PB vs NIC (n = 37), PB vs TIC (n = 36), and NIC vs TIC (n = 38). Each dot represents one patient. Correlations between paired data were analyzed using Pearson's correlation coefficient.

Supplementary Figure S3

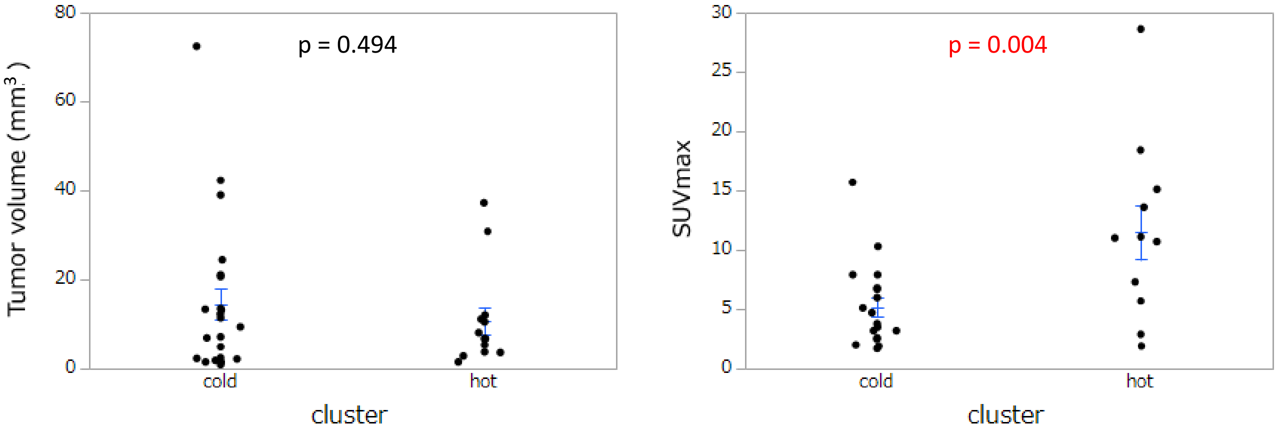

Supplementary Figure S3. Relationships among clinical characteristics and immune profiling in lung tumor tissues. Tumor volume (n = 36) and SUVmax (n = 30) were compared between immunologically "hot" and "cold" clusters. Tumor volume (V) was calculated using the following formula: V = length × width × thickness / 2. Measurements were performed directly using surgically resected specimens. Each dot represents one patient. The mean ± SEM was shown. An unpaired two-tailed Student's *t*-test was used to examine the significance of differences between samples, with a p value < 0.05 indicating a significant difference.

Supplementary Figure S4

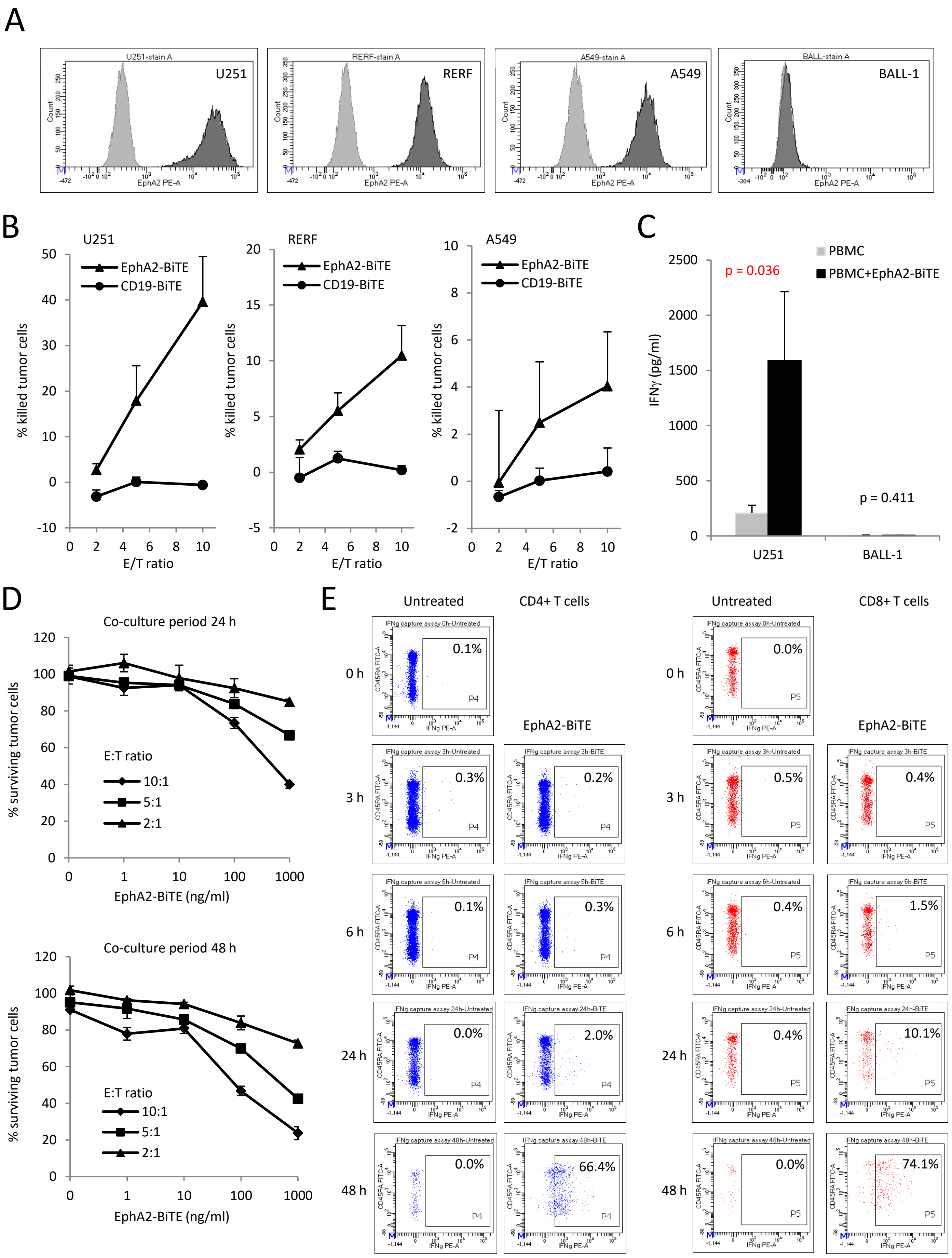

Supplementary Figure S4. Optimization of the assay system for T-cell cytotoxicity. A) Expression of EphA2 in tumor cell lines. The expression of EphA2 in U251, RERF-LC-AI, A549, and BALL-1 cell lines was measured by flow cytometry (gray-filled curve: isotype, black-filled curve: anti-EphA2-PE). RERF-LC-AI, A549, and BALL-1 cell lines were purchased from the RIKEN BioResource Center. Anti-EphA2-PE (clone SHM16) was purchased from Biolegend. B) Cytotoxicity of the EphA2/CD3 bispecific T-cell engager (EphA2-BiTE) to various tumor cell lines. Cytotoxic activity against U251, RERF-LC-AI, or A549 cells in a co-culture of healthy donor PBMC (E/T ratio of 2:1, 5:1, or 10:1) with EphA2-BiTE (100 ng/ml) was analyzed after 48 hours using a MTS assay (n = 3). The CD19/CD3 bispecific T-cell engager (CD19-BiTE, G&P Biosciences) was used as a control. Data represent the mean ± SEM. C) The specificity of EphA2-BiTE. IFNγ production after a 24-hour co-culture of U251 (EphA2-positive cells) and healthy donor PBMC (n = 3, E/T ratio of 5:1) with or without EphA2-BiTE (100 ng/ml) was measured using ELISA as per the manufacturer's instructions (R&D Systems). BALL-1 (EphA2 negative cells) was used as a control. The mean ± SEM was shown. A paired one-tailed Student's *t*-test was used to examine the significance of differences between samples, with a p value < 0.05 indicating a significant difference. D) Optimization of co-culture conditions. Cytotoxic activity against U251 cells was analyzed under different E/T ratios, EphA2-BiTE concentrations, and culture periods. Data represent the mean ± S.D. of triplicate experiments. E) IFNγ secretion from $CD4^+$ and $CD8^+$ T cells in a co-culture of U251 and healthy donor PBMC (E/T ratio of 5:1) with EphA2-BiTE (100 ng/ml) was analyzed at various time periods (0, 3, 6, 24, and 48 hours).

Supplementary Figure S5

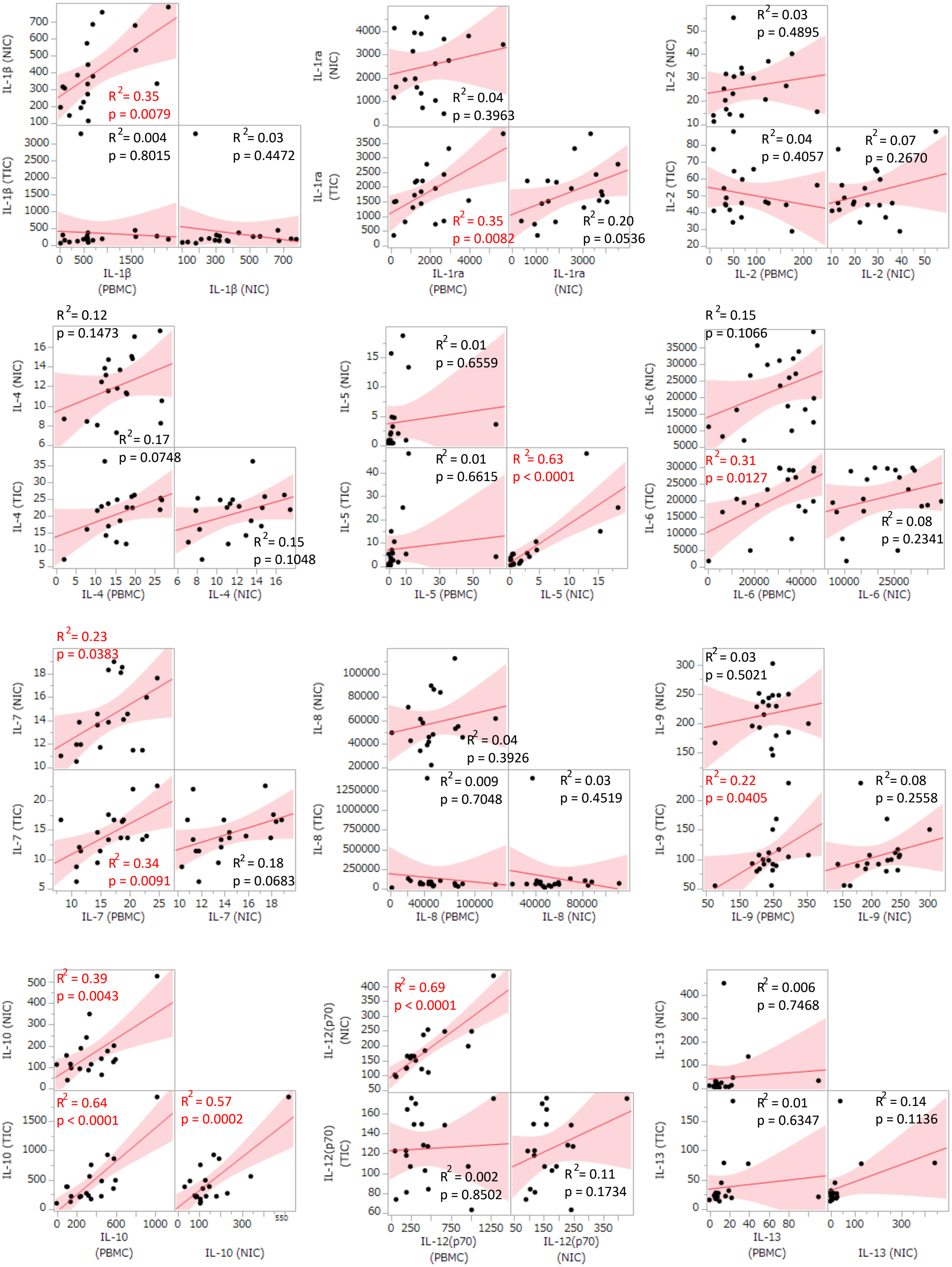

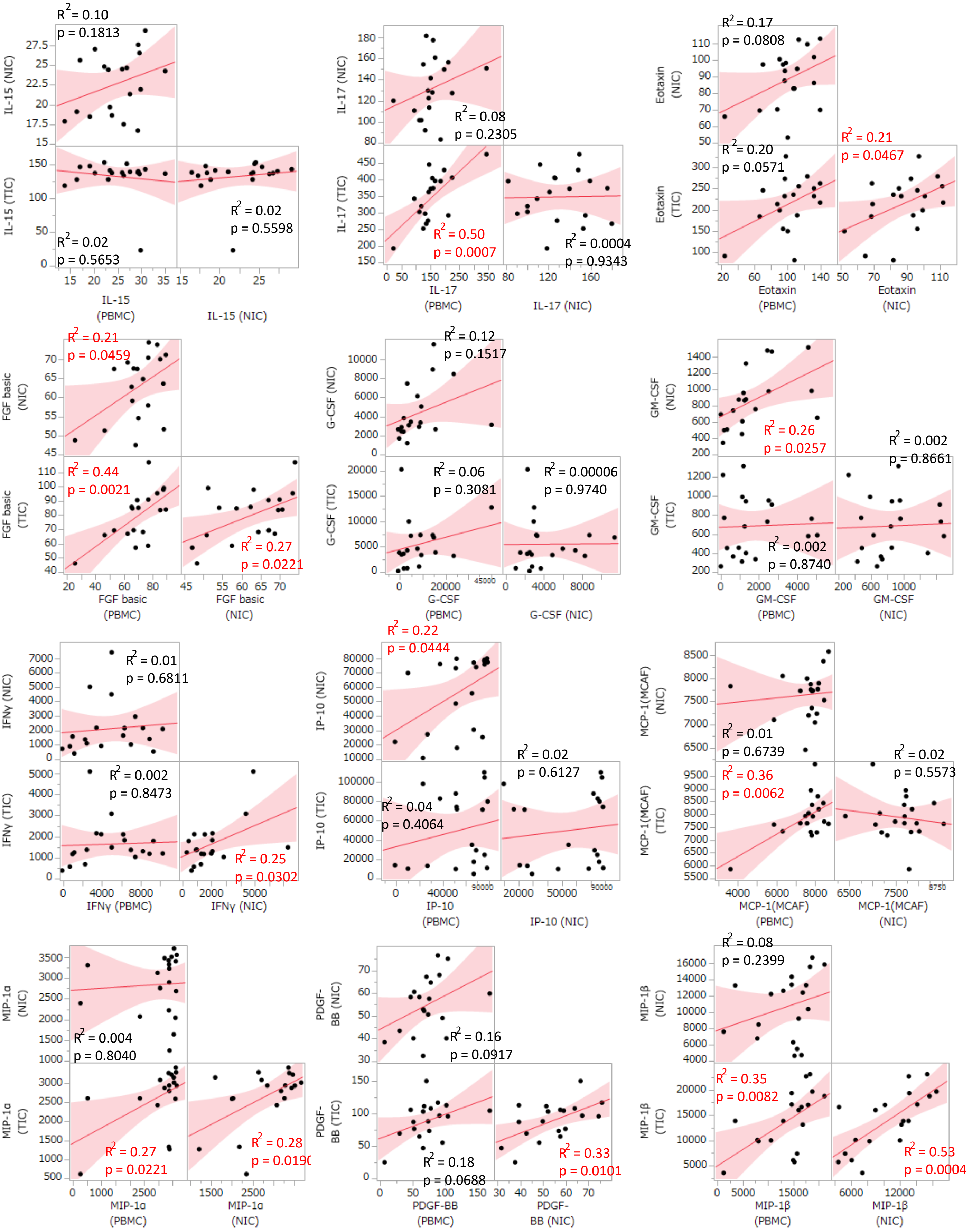

IL-15 (NIC)
IL-15 (TIC)
IL-15 (PBMC)
IL-15 (NIC)
R2 = 0.10
p = 0.1813
R2 = 0.02
p = 0.5653
R2 = 0.02
p = 0.5598
IL-17 (NIC)
IL-17 (TIC)
IL-17 (PBMC)
IL-17 (NIC)
R2 = 0.08
p = 0.2305
R2 = 0.50
p = 0.0007
R2 = 0.0004
p = 0.9343
Eotaxin (NIC)
Eotaxin (TIC)
Eotaxin (PBMC)
Eotaxin (NIC)
R2 = 0.17
p = 0.0808
R2 = 0.20
p = 0.0571
R2 = 0.21
p = 0.0467
FGF basic (NIC)
FGF basic (TIC)
FGF basic (PBMC)
FGF basic (NIC)
R2 = 0.21
p = 0.0459
R2 = 0.44
p = 0.0021
R2 = 0.27
p = 0.0221
G-CSF (NIC)
G-CSF (TIC)
G-CSF (PBMC)
G-CSF (NIC)
R2 = 0.12
p = 0.1517
R2 = 0.06
p = 0.3081
R2 = 0.00006
p = 0.9740
GM-CSF (NIC)
GM-CSF (TIC)
GM-CSF (PBMC)
GM-CSF (NIC)
R2 = 0.26
p = 0.0257
R2 = 0.002
p = 0.8661
R2 = 0.002
p = 0.8740
IFNγ (NIC)
IFNγ (TIC)
IFNγ (PBMC)
IFNγ (NIC)
R2 = 0.01
p = 0.6811
R2 = 0.002
p = 0.8473
R2 = 0.25
p = 0.0302
IP-10 (NIC)
IP-10 (TIC)
IP-10 (PBMC)
IP-10 (NIC)
R2 = 0.22
p = 0.0444
R2 = 0.04
p = 0.4064
R2 = 0.02
p = 0.6127
MCP-1(MCAF) (NIC)
MCP-1(MCAF) (TIC)
MCP-1(MCAF) (PBMC)
MCP-1(MCAF) (NIC)
R2 = 0.01
p = 0.6739
R2 = 0.36
p = 0.0062
R2 = 0.02
p = 0.5573
MIP-1α (NIC)
MIP-1α (TIC)
MIP-1α (PBMC)
MIP-1α (NIC)
R2 = 0.004
p = 0.8040
R2 = 0.27
p = 0.0221
R2 = 0.28
p = 0.0190
PDGF-BB (NIC)
PDGF-BB (TIC)
PDGF-BB (PBMC)
PDGF-BB (NIC)
R2 = 0.16
p = 0.0917
R2 = 0.18
p = 0.0688
R2 = 0.33
p = 0.0101
MIP-1β (NIC)
MIP-1β (TIC)
MIP-1β (PBMC)
MIP-1β (NIC)
R2 = 0.08
p = 0.2399
R2 = 0.35
p = 0.0082
R2 = 0.53
p = 0.0004

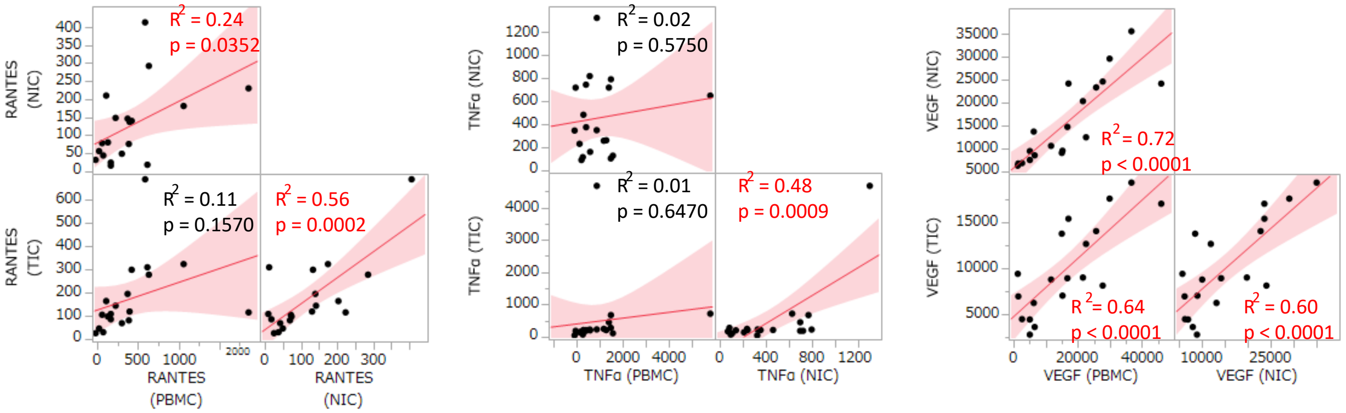

Supplementary Figure S5. Relationships of cytokine production among PBMC, normal lung tissues, and tumor tissues (n = 19). Correlations between paired data were analyzed using Pearson's correlation coefficient. Each dot represents one patient. The unit for the concentration of cytokines is pg/ml.

Supplementary Figure S6

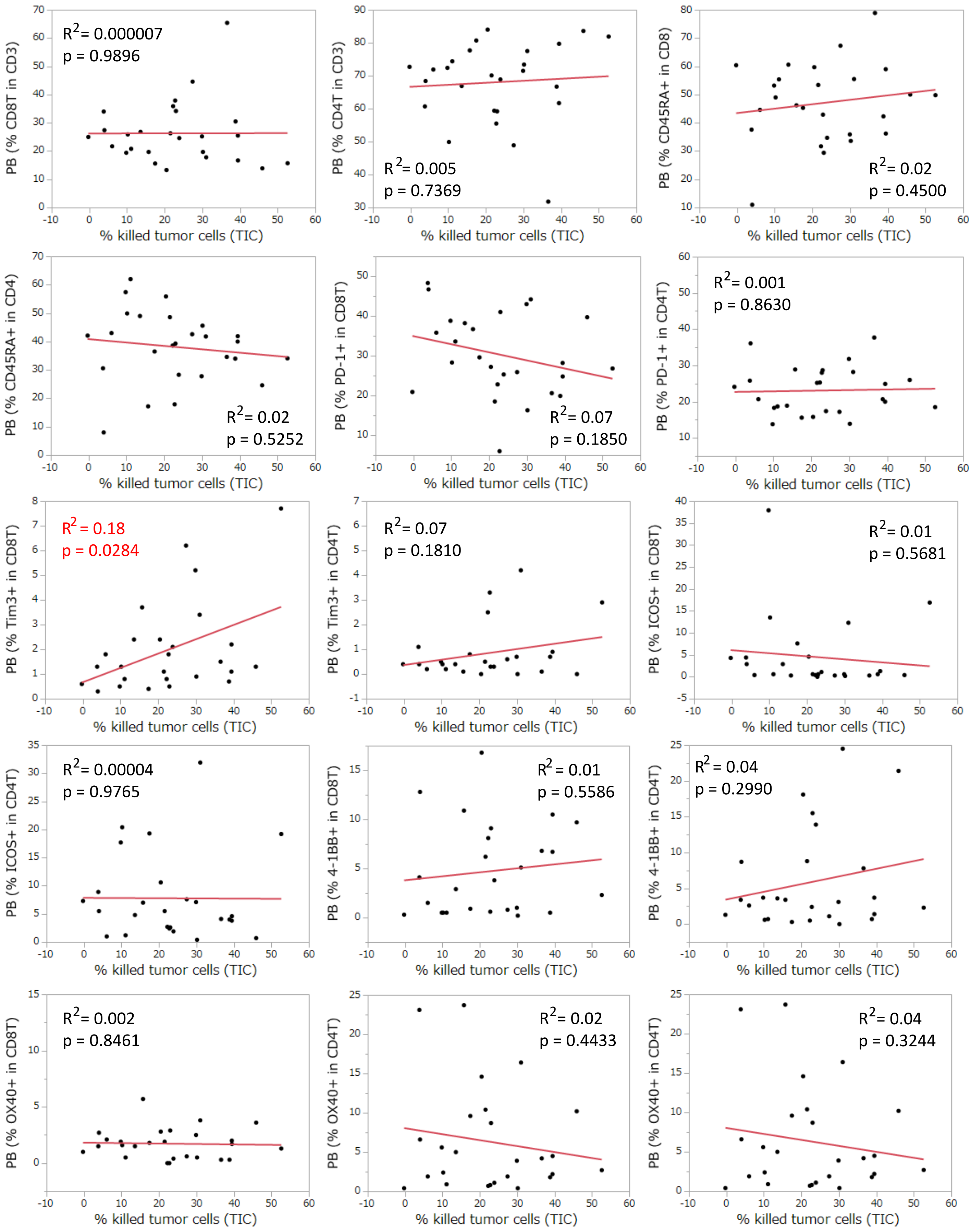

Supplementary Figure S6. Relationship between the immune profile in peripheral blood and the cytotoxic activity of T cells in lung tumor tissues. Fifteen factors of the immune profile in peripheral blood (PB) were analyzed for their relationships with the cytotoxic activity of T cells in lung tumor-infiltrated cells (TIC) (n = 26). Each dot represents one patient. Correlations between paired data were analyzed using Pearson’s correlation coefficient.

Supplementary Figure S7

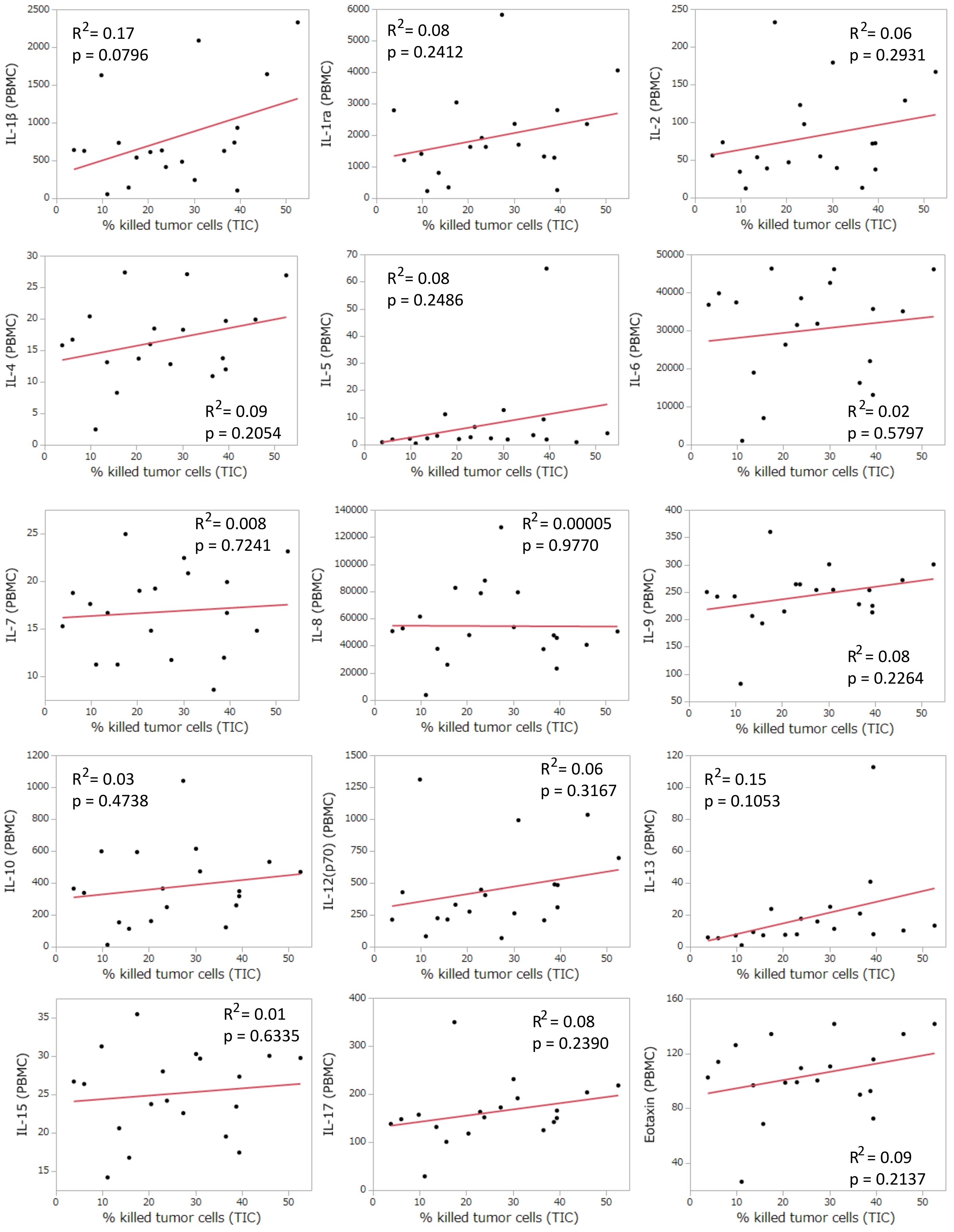

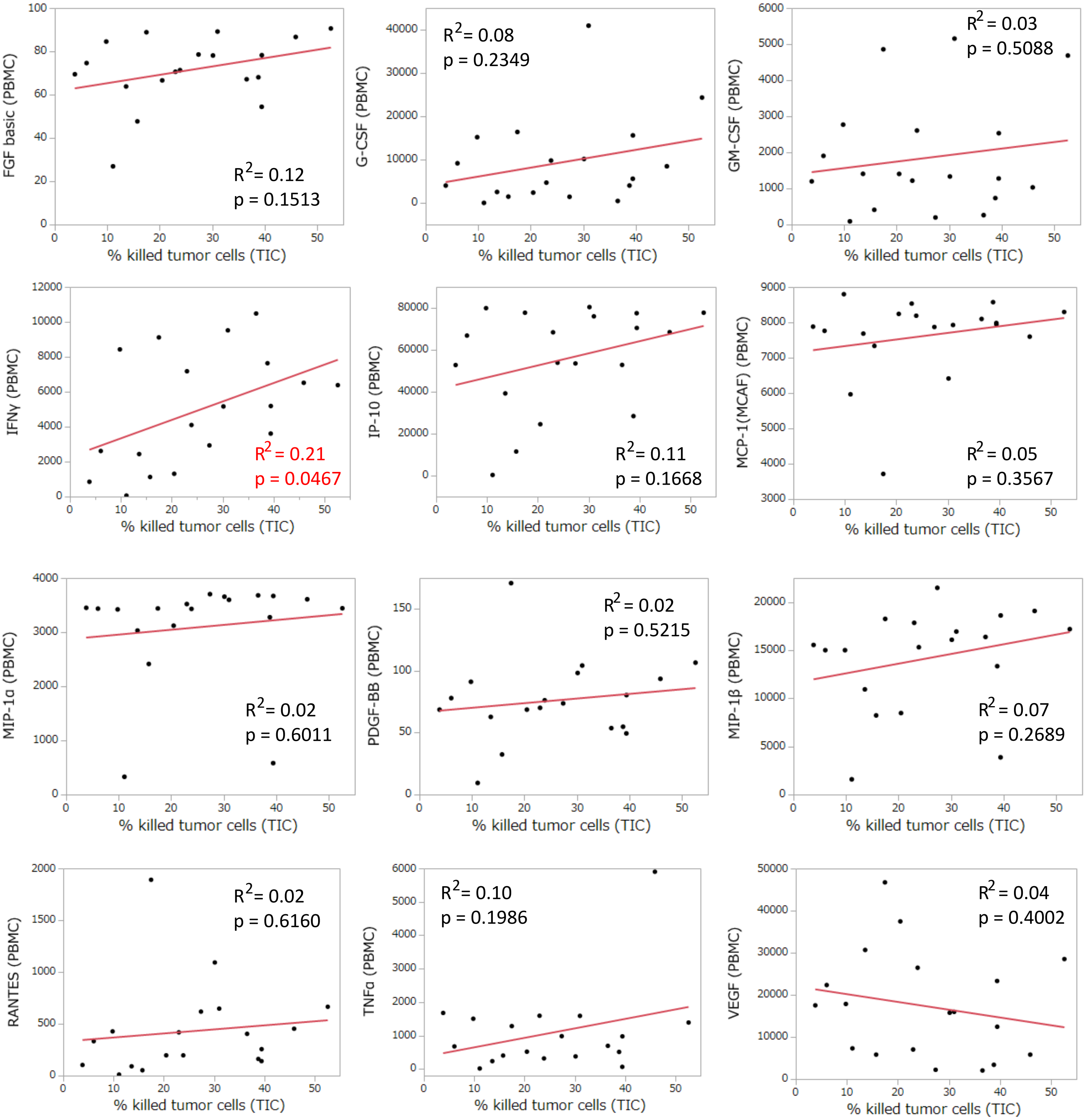
FGF basic (PBMC)
R2= 0.12
p = 0.1513
% killed tumor cells (TIC)
G-CSF (PBMC)
R2= 0.08
p = 0.2349
% killed tumor cells (TIC)
GM-CSF (PBMC)
R2 = 0.03
p = 0.5088
% killed tumor cells (TIC)
IFNγ (PBMC)
R2 = 0.21
p = 0.0467
% killed tumor cells (TIC)
IP-10 (PBMC)
R2= 0.11
p = 0.1668
% killed tumor cells (TIC)
MCP-1(MCAF) (PBMC)
R2 = 0.05
p = 0.3567
% killed tumor cells (TIC)
MIP-1α (PBMC)
R2= 0.02
p = 0.6011
% killed tumor cells (TIC)
PDGF-BB (PBMC)
R2 = 0.02
p = 0.5215
% killed tumor cells (TIC)
MIP-1β (PBMC)
R2 = 0.07
p = 0.2689
% killed tumor cells (TIC)
RANTES (PBMC)
R2= 0.02
p = 0.6160
% killed tumor cells (TIC)
TNFα (PBMC)
R2= 0.10
p = 0.1986
% killed tumor cells (TIC)
VEGF (PBMC)
R2 = 0.04
p = 0.4002
% killed tumor cells (TIC)

Supplementary Figure S7. Relationship between cytokine production in PBMC and the cytotoxic activity of T cells in lung tumor tissues. Twenty-seven cytokines in a co-culture with PBMC were analyzed for their relationships with the cytotoxic activity of T cells in lung tumor-infiltrated cells (TIC) (n = 19). Each dot represents one patient. Correlations between paired data were analyzed using Pearson's correlation coefficient. The unit for the concentration of cytokines is pg/ml.

Supplementary Figure S8

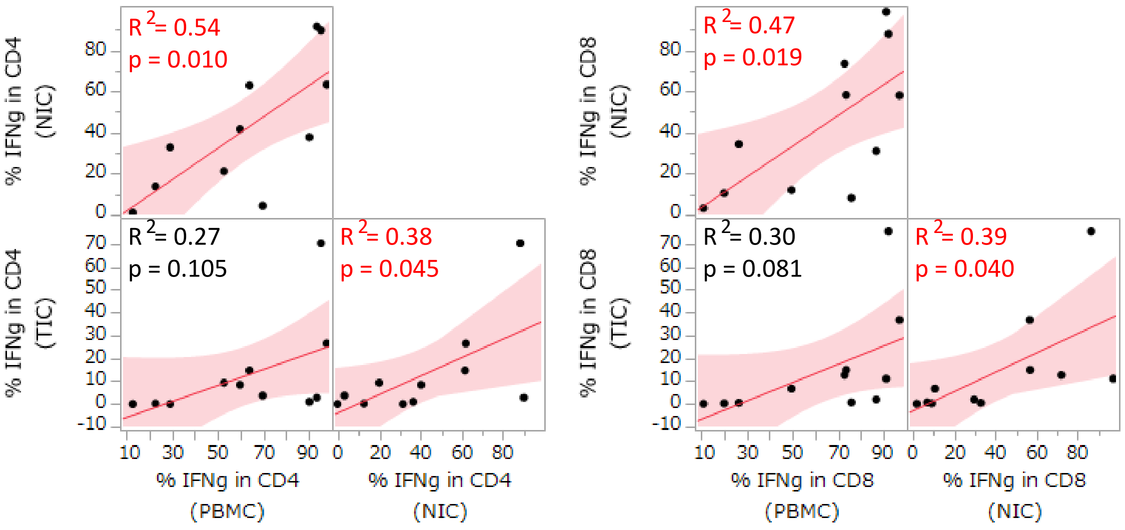

Supplementary Figure S8. IFNγ production from CD4⁺ and CD8⁺ T cells in PBMC, normal lung tissues, and tumor tissues. IFNg production from $CD4^+$ and $CD8^+$ T cells in PBMC, normal lung tissues, and tumor tissues. IFNg production by $CD4^+$ and $CD8^+$ T cells was analyzed using the IFNg secretion assay in co-culture experiments (n = 11). The ratio of IFNg-producing cells was analyzed by FACS. Each dot represents one patient. Correlations between paired data were analyzed using Pearson's correlation coefficient.

Supplementary Figure S9

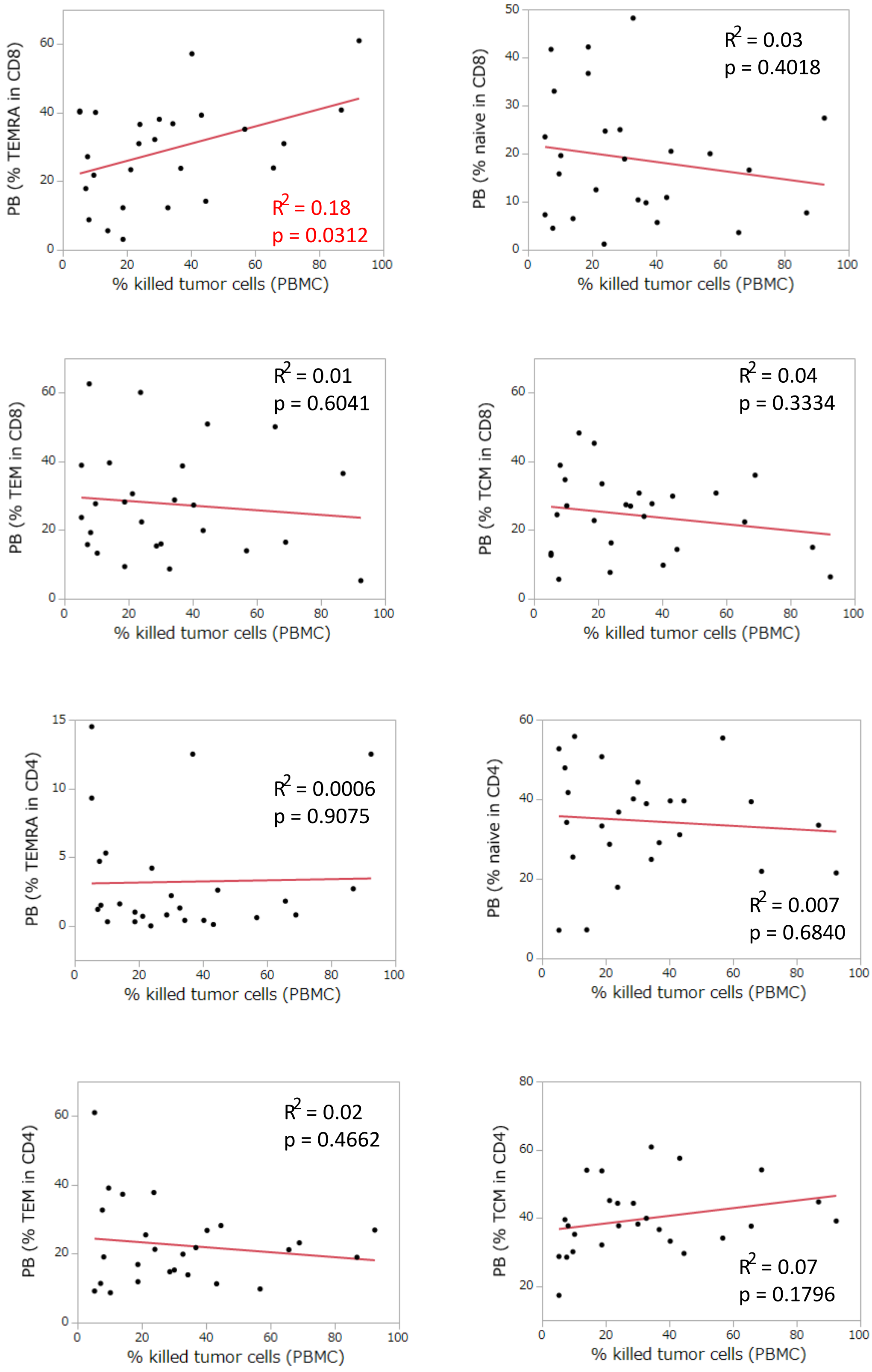

Supplementary Figure S9. Relationship between the phenotype of peripheral T cells and T cell cytotoxicity in peripheral blood. The ratio of naïve ($CD27^+$ and $CD45RA^+$), central memory (TCM, $CD27^+/CD45RA^-$), effector memory (TEM, $CD27^-/CD45RA^-$), and effector memory re-expresses CD45RA (TEMRA, $CD45RA^+/CD27^-$) in $CD4^+$ and $CD8^+$ T cells was analyzed for correlations with the cytotoxic activity of T cells in PBMC. Correlations between paired data were analyzed using Pearson’s correlation coefficient.

Supplementary Figure S10

A TCRα

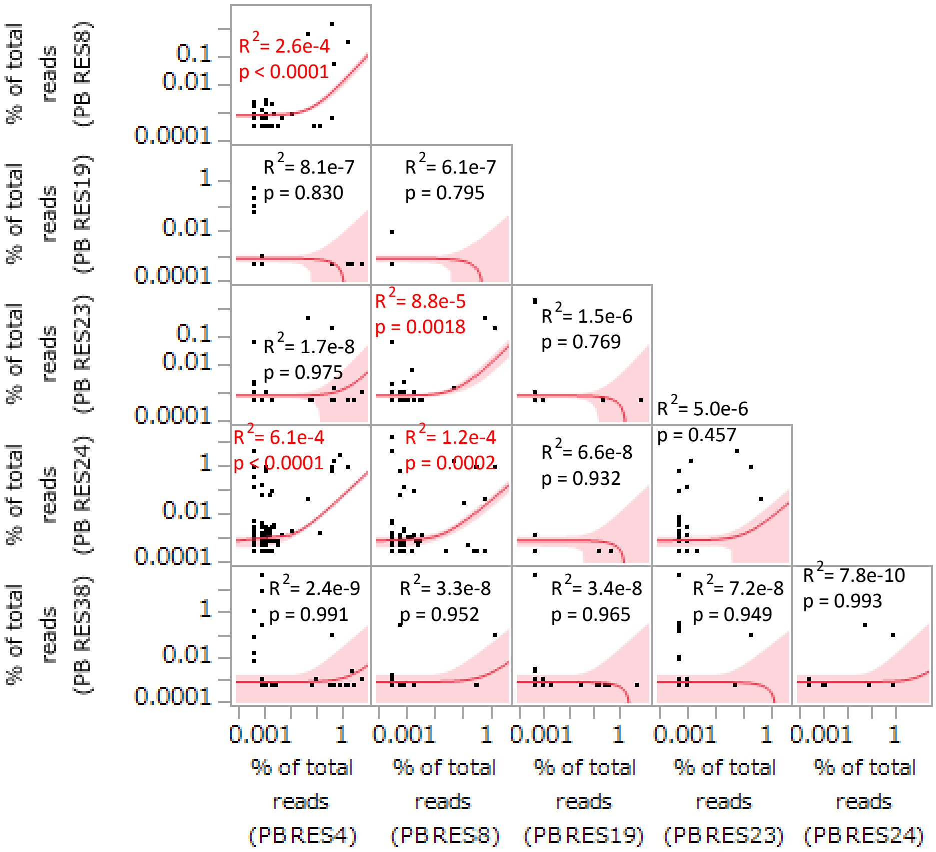

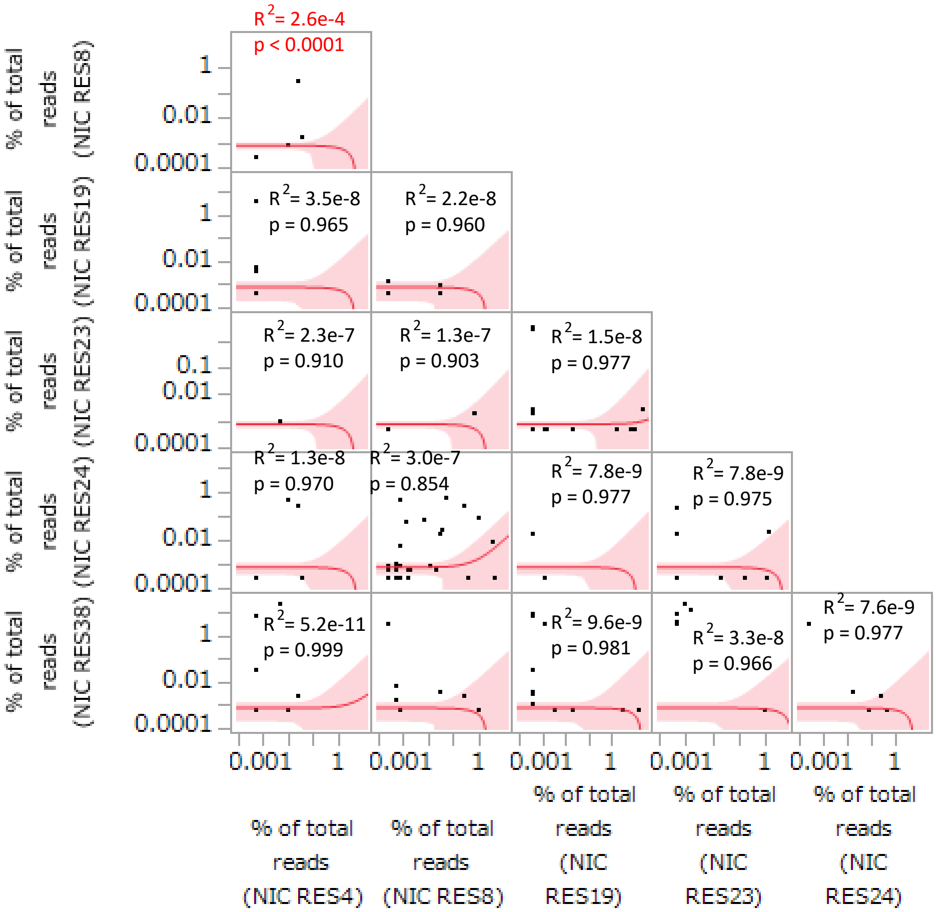

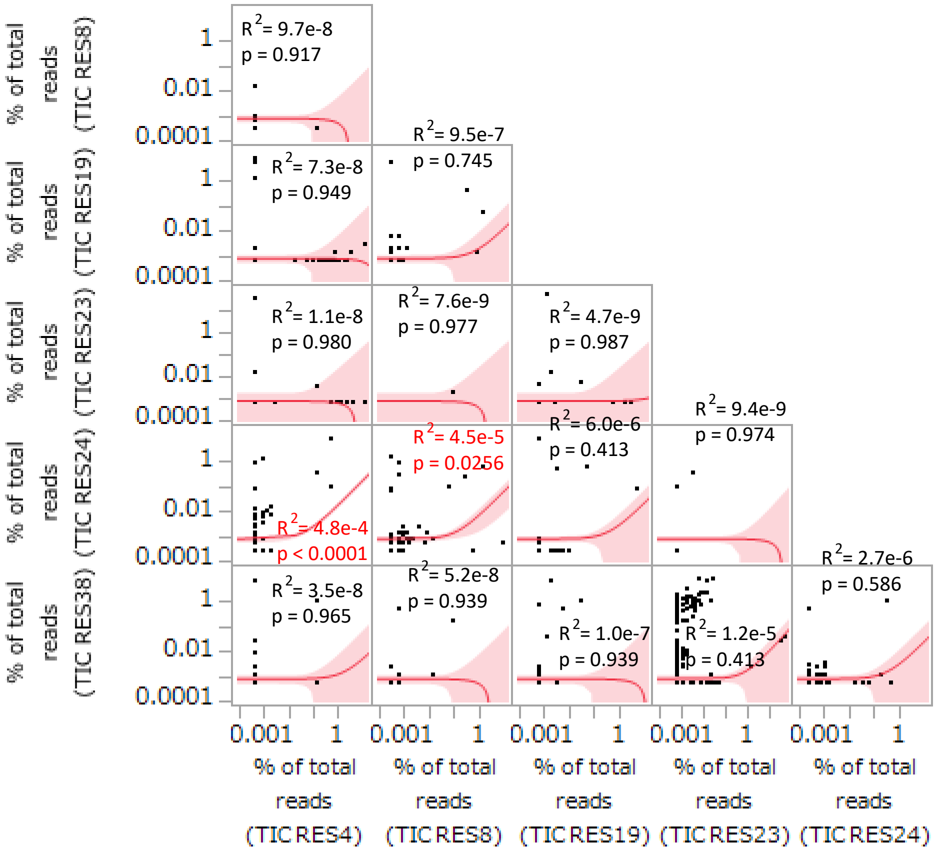

R 2= 9.7e-8
p = 0.917
R 2= 9.5e-7
p = 0.745
R 2= 7.3e-8
p = 0.949
R 2= 1.1e-8
p = 0.980
R 2= 7.6e-9
p = 0.977
R 2= 4.7e-9
p = 0.987
R 2= 9.4e-9
p = 0.974
R 2= 4.5e-5
p = 0.0256
R 2= 6.0e-6
p = 0.413
R 2= 4.8e-4
p < 0.0001
R 2= 2.7e-6
p = 0.586
R 2= 3.5e-8
p = 0.965
R 2= 5.2e-8
p = 0.939
R 2= 1.0e-7
p = 0.939
R 2= 1.2e-5
p = 0.413
1
0.01
0.0001
0.001
% of total reads (TIC RES8)
% of total reads (TIC RES19)
% of total reads (TIC RES23)
% of total reads (TIC RES24)
% of total reads (TIC RES38)
% of total reads (TIC RES4)
% of total reads (TIC RES8)
% of total reads (TIC RES19)
% of total reads (TIC RES23)
% of total reads (TIC RES24)

B TCRβ

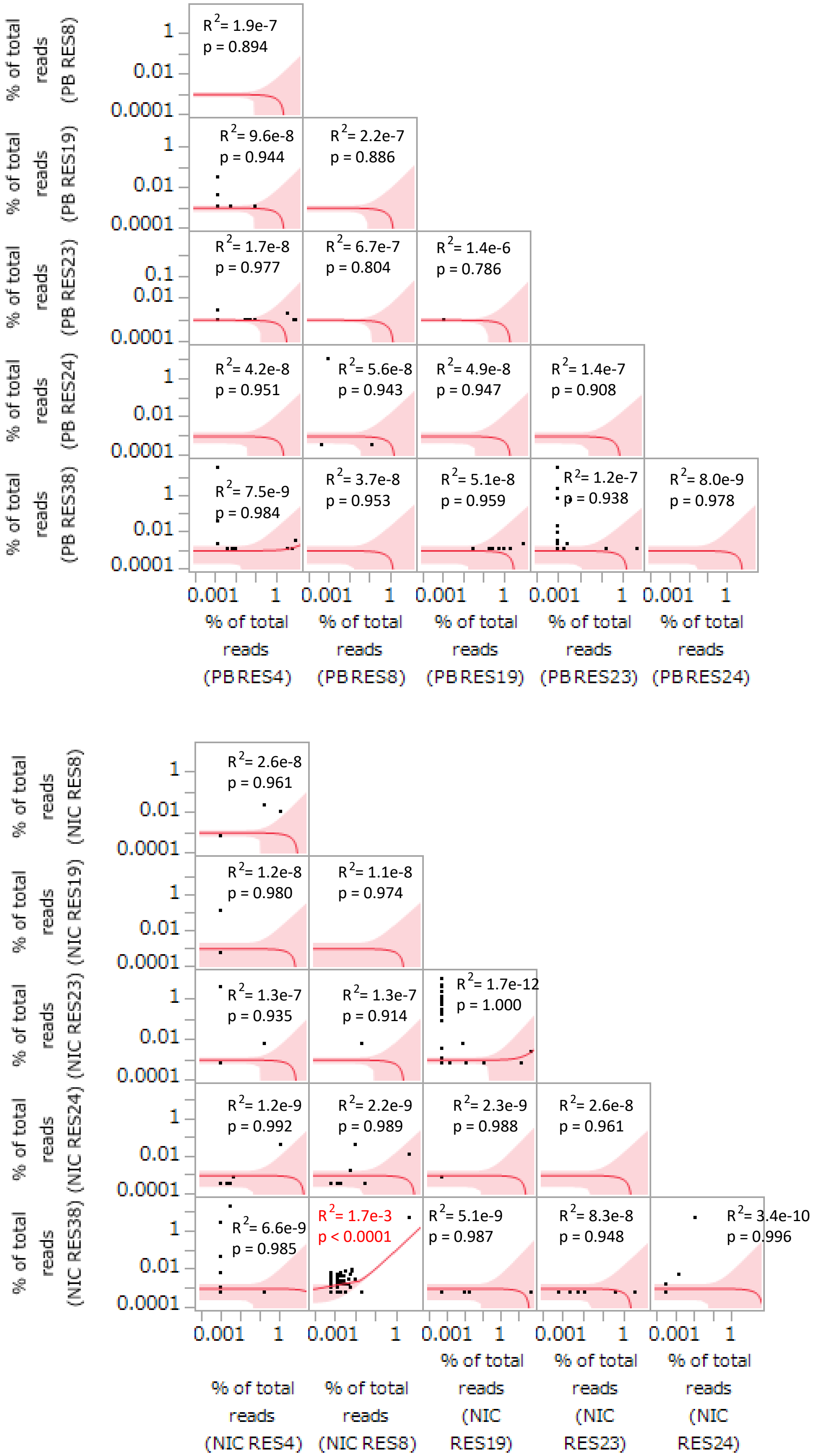

% of total reads (PB RES8)
% of total reads (PB RES19)
% of total reads (PB RES23)
% of total reads (PB RES24)
% of total reads (PB RES38)
R2= 1.9e-7 p = 0.894
R2= 9.6e-8 p = 0.944
R2= 2.2e-7 p = 0.886
R2= 1.7e-8 p = 0.977
R2= 6.7e-7 p = 0.804
R2= 1.4e-6 p = 0.786
R2= 4.2e-8 p = 0.951
R2= 5.6e-8 p = 0.943
R2= 4.9e-8 p = 0.947
R2= 1.4e-7 p = 0.908
R2= 7.5e-9 p = 0.984
R2= 3.7e-8 p = 0.953
R2= 5.1e-8 p = 0.959
R2= 1.2e-7 p = 0.938
R2= 8.0e-9 p = 0.978
% of total reads (PB RES4)
% of total reads (PB RES8)
% of total reads (PB RES19)
% of total reads (PB RES23)
% of total reads (PB RES24)
% of total reads (NIC RES8)
% of total reads (NIC RES19)
% of total reads (NIC RES23)
% of total reads (NIC RES24)
% of total reads (NIC RES38)
R2= 2.6e-8 p = 0.961
R2= 1.2e-8 p = 0.980
R2= 1.1e-8 p = 0.974
R2= 1.3e-7 p = 0.935
R2= 1.3e-7 p = 0.914
R2= 1.7e-12 p = 1.000
R2= 1.2e-9 p = 0.992
R2= 2.2e-9 p = 0.989
R2= 2.3e-9 p = 0.988
R2= 2.6e-8 p = 0.961
R2= 6.6e-9 p = 0.985
R2= 1.7e-3 p < 0.0001
R2= 5.1e-9 p = 0.987
R2= 8.3e-8 p = 0.948
R2= 3.4e-10 p = 0.996
% of total reads (NIC RES4)
% of total reads (NIC RES8)
% of total reads (NIC RES19)
% of total reads (NIC RES23)
% of total reads (NIC RES24)

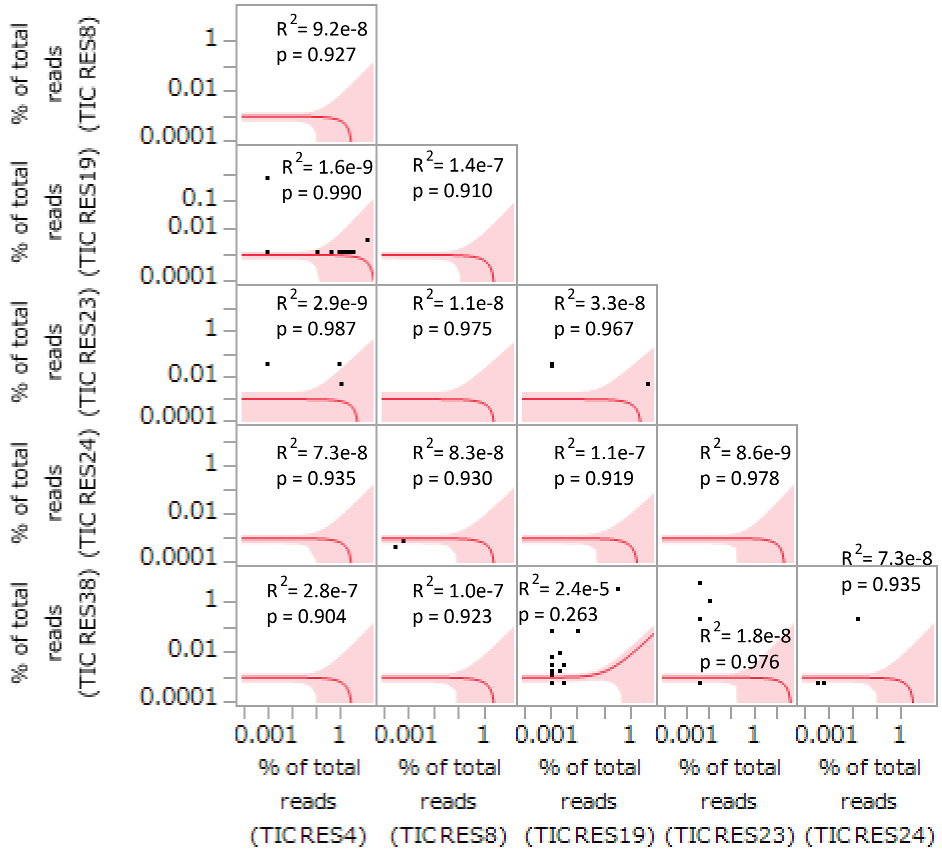

R 2= 9.2e-8
p = 0.927
R 2= 1.6e-9
p = 0.990
R 2= 1.4e-7
p = 0.910
R 2= 2.9e-9
p = 0.987
R 2= 1.1e-8
p = 0.975
R 2= 3.3e-8
p = 0.967
R 2= 7.3e-8
p = 0.935
R 2= 8.3e-8
p = 0.930
R 2= 1.1e-7
p = 0.919
R 2= 8.6e-9
p = 0.978
R 2= 2.8e-7
p = 0.904
R 2= 1.0e-7
p = 0.923
R 2= 2.4e-5
p = 0.263
R 2= 1.8e-8
p = 0.976
R 2= 7.3e-8
p = 0.935
% of total reads (TIC RES8)
% of total reads (TIC RES19)
% of total reads (TIC RES23)
% of total reads (TIC RES24)
% of total reads (TIC RES38)
% of total reads (TIC RES4)
% of total reads (TIC RES8)
% of total reads (TIC RES19)
% of total reads (TIC RES23)
% of total reads (TIC RES24)
1
0.1
0.01
0.0001
0.001

Supplementary Figure S10. Clonotypes of $CD8^+$ T cells from PBMC, normal lung tissues, and tumor tissues were compared among patients. Correlations between paired data were analyzed using Pearson's correlation coefficient.

Supplementary Figure S11

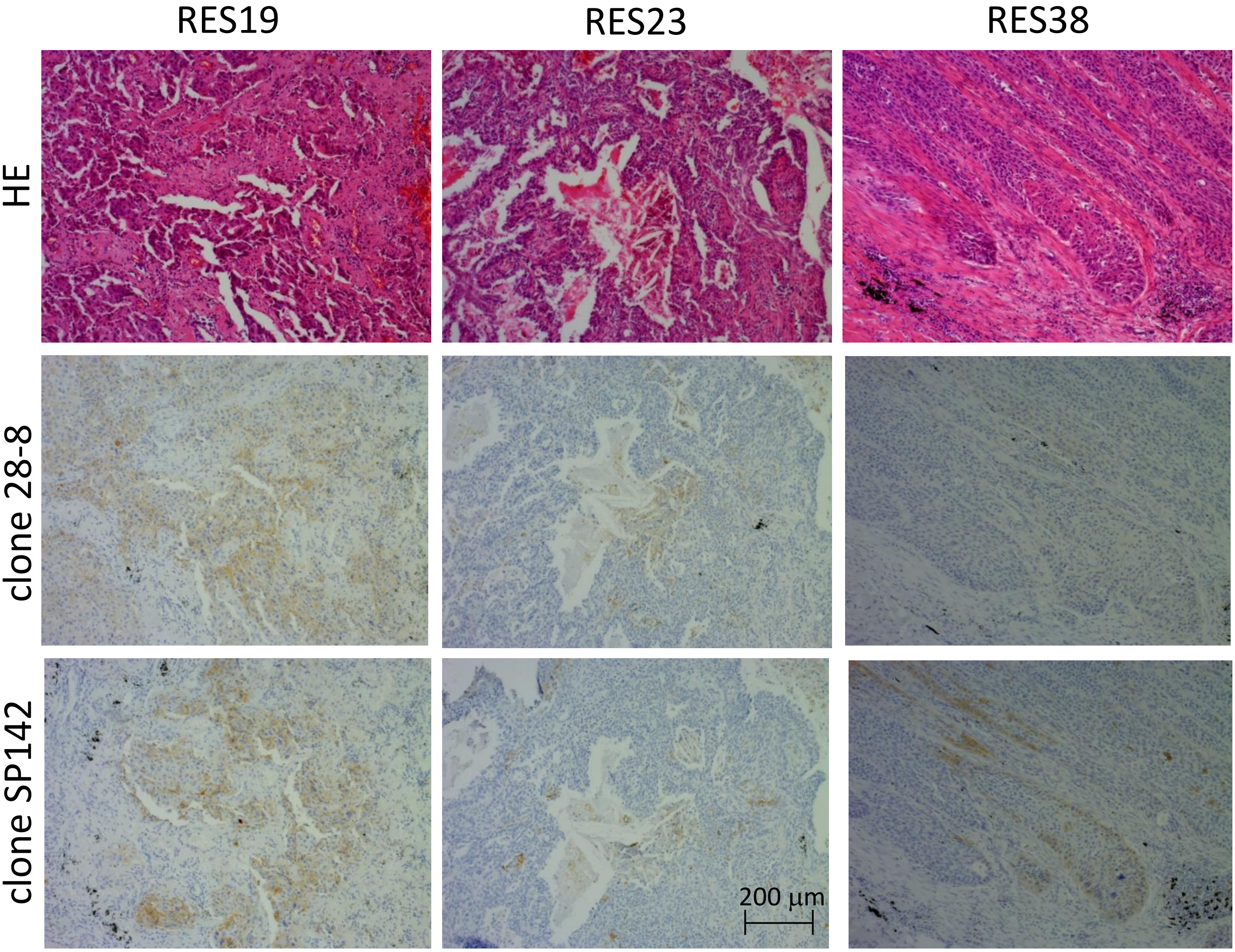

Supplementary Figure S11. PD-L1 expression on tumor cells in NSCLC patients. PD-L1 expression on tumor cells was analyzed by immunohistochemistry using anti-PD-L1 antibodies (clone 28-8 and clone SP142). Hematoxylin and eosin (HE) staining was also shown. PD-L1 staining of patients with strong (RES38), moderate (RES19), and weak (RES23) cytotoxicities was indicated.

Supplementary Figure S12

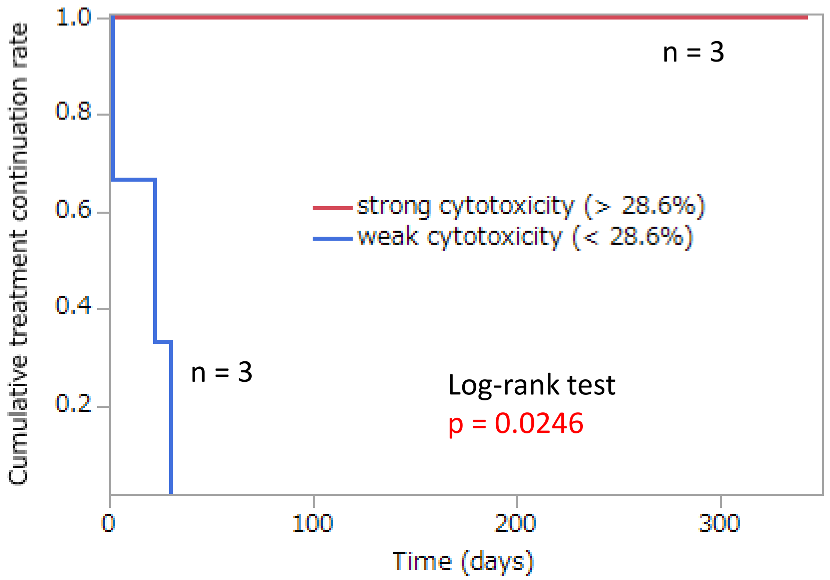

Supplementary Figure S12. Peripheral T cell cytotoxicity in PD-1 inhibitor recipients. Peripheral T cell cytotoxicity was analyzed before the PD-1 inhibitor treatment (pembrolizumab: n = 4, nivolumab: n = 2). A Kaplan-Meier analysis was used to evaluate the rate of continuation since the initiation of treatment. Patients with strong (> 28.6%, n = 3) and weak (< 28.6%, n = 3) cytotoxicities were compared by the Log-rank test with a p value < 0.05 indicating a significant difference.

Supplementary Figure S13

TIC lymphocytes (RES29)

	FITC	PerCP eFluor 710	PE	PE CF594	PE Cy7	BV421	BV510	BV605	BV711	BV786	APC	Alexa Fluor 700	APC Cy7
TIC lympho	CD45RA	ICOS	CD25	OX40	PD-1	4-1BB	CD8	CD103	CD4	CD45	Tim-3	CD3	Live/Dead
TIC lympho iso	CD45RA	iso	iso	iso	iso	iso	CD8	iso	CD4	CD45	iso	CD3	Live/Dead

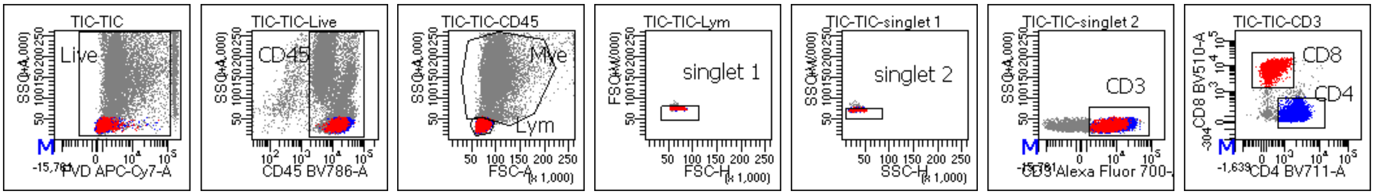

CD4+ T cells

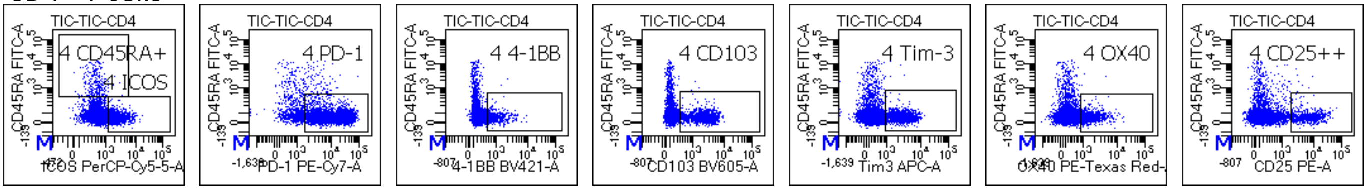

CD8+ T cells

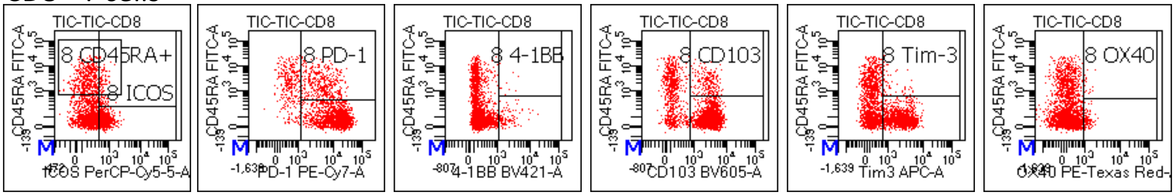

TIC lymphocyte isotype-control (RES29)

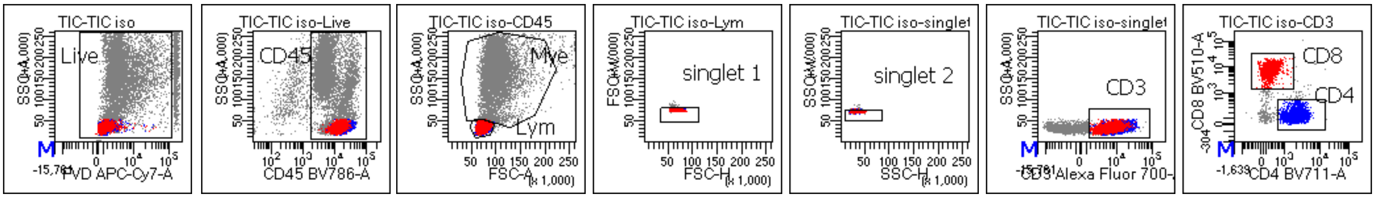

CD4+ T cells

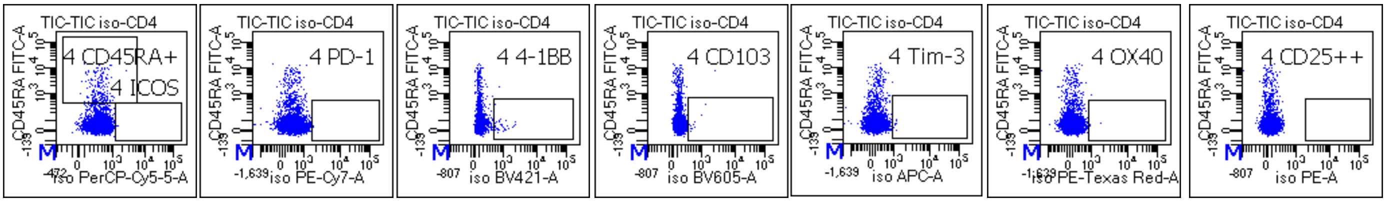

CD8+ T cells

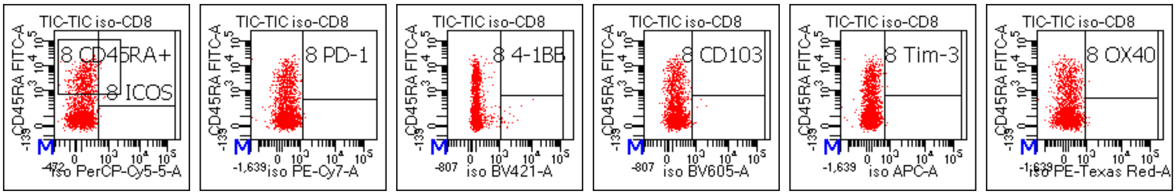

Tube: TIC

Population	#Events	%Parent	%Total
All Events	27,998	####	100.0
Live	20,132	71.9	71.9
CD45	19,330	96.0	69.0
Lym	9,457	48.9	33.8
singlet 1	9,429	99.7	33.7
singlet 2	9,372	99.4	33.5
CD3	7,402	79.0	26.4
CD4	4,507	60.9	16.1
4 CD45RA+	246	5.5	0.9
4 CD25++	612	13.6	2.2
4 ICOS	1,047	23.2	3.7
4 PD-1	3,647	80.9	13.0
4 4-1BB	422	9.4	1.5
4 CD103	1,181	26.2	4.2
4 Tim-3	1,223	27.1	4.4
4 OX40	896	19.9	3.2
CD8	2,492	33.7	8.9
8 ICOS	743	29.8	2.7
8 PD-1	2,172	87.2	7.8
8 4-1BB	107	4.3	0.4
8 CD103	1,782	71.5	6.4
8 Tim-3	1,147	46.0	4.1
8 OX40	116	4.7	0.4
8 CD45RA+	551	22.1	2.0
Mye	9,565	49.5	34.2

Tube: TIC iso

Population	#Events	%Parent	%Total
All Events	21,383	####	100.0
Live	15,301	71.6	71.6
CD45	14,699	96.1	68.7
Lym	7,361	50.1	34.4
singlet 1	7,329	99.6	34.3
singlet 2	7,299	99.6	34.1
CD3	5,790	79.3	27.1
CD4	3,546	61.2	16.6
4 CD45RA+	195	5.5	0.9
4 CD25++	0	0.0	0.0
4 ICOS	5	0.1	0.0
4 PD-1	3	0.1	0.0
4 4-1BB	33	0.9	0.2
4 CD103	13	0.4	0.1
4 Tim-3	7	0.2	0.0
4 OX40	3	0.1	0.0
CD8	1,963	33.9	9.2
8 ICOS	8	0.4	0.0
8 PD-1	4	0.2	0.0
8 4-1BB	17	0.9	0.1
8 CD103	6	0.3	0.0
8 Tim-3	6	0.3	0.0
8 OX40	6	0.3	0.0
8 CD45RA+	426	21.7	2.0
Mye	7,097	48.3	33.2

TIC Myeloid cells (RES29)

	PE CF594	BV510	BV605	BV711	BV786	Alexa Fluor 700	APC Cy7
TIC Myelo	CCR3	CD15	CD11b	CD33	CD45	CD14	Live/Dead

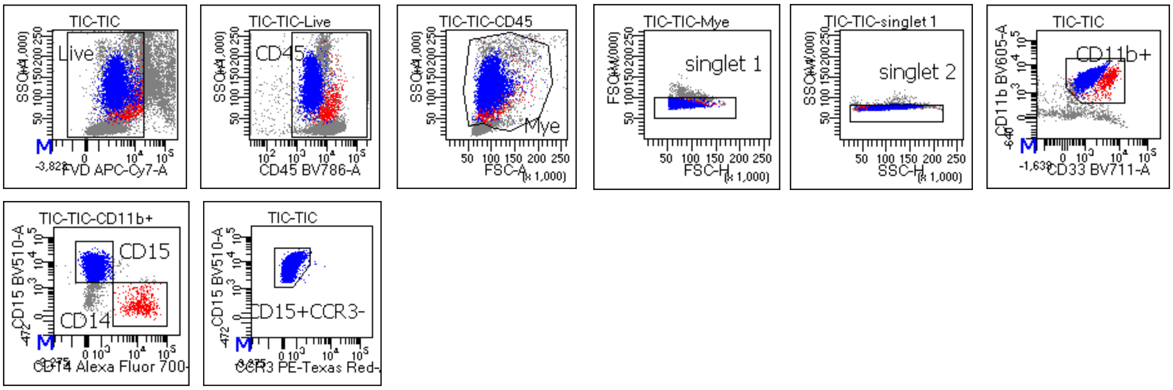

Tube: TIC

Population	#Events	%Parent	%Total
All Events	25,016	####	100.0
Live	19,778	79.1	79.1
CD45	16,221	82.0	64.8
Mye	6,902	42.5	27.6
singlet 1	6,547	94.9	26.2
singlet 2	6,383	97.5	25.5
CD11b+	5,569	87.2	22.3
CD14	505	9.1	2.0
CD15	4,446	79.8	17.8
CD15+CCR3-	4,430	99.6	17.7

NIC lymphocytes (RES29)

	FITC	PerCP eFluor 710	PE	PE CF594	PE Cy7	BV421	BV510	BV605	BV711	BV786	APC	Alexa Fluor 700	APC Cy7
NIC lympho	CD45RA	ICOS	CD25	OX40	PD-1	4-1BB	CD8	CD103	CD4	CD45	Tim-3	CD3	Live/Dead
NIC lympho iso	CD45RA	iso	iso	iso	iso	iso	CD8	iso	CD4	CD45	iso	CD3	Live/Dead

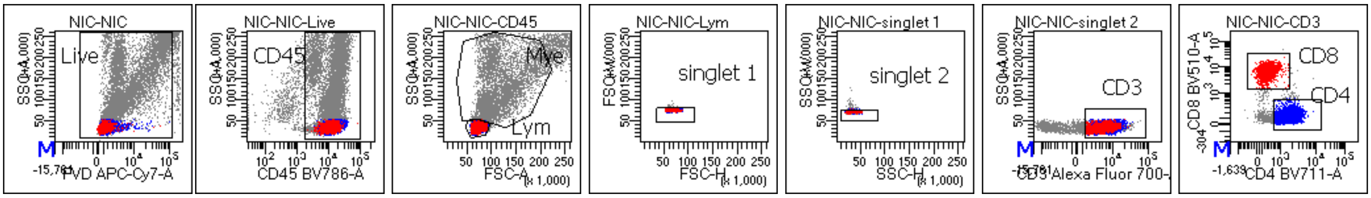

CD4+ T cells

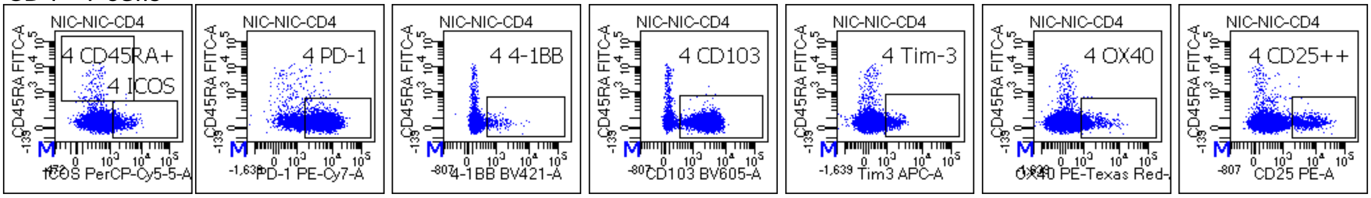

CD8+ T cells

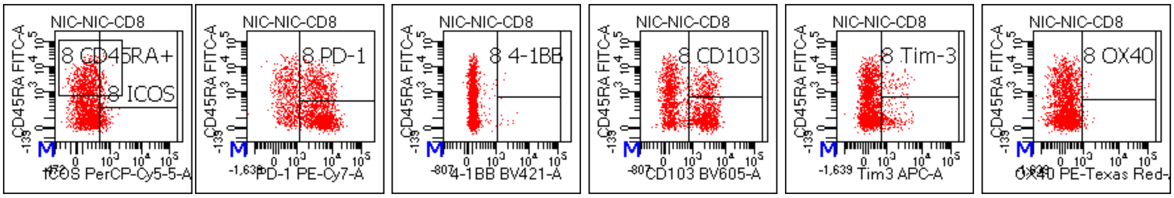

NIC lymphocyte isotype-control (RES29)

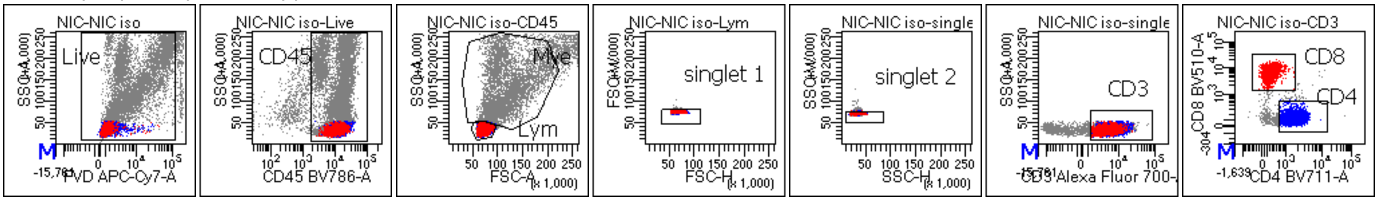

CD4+ T cells

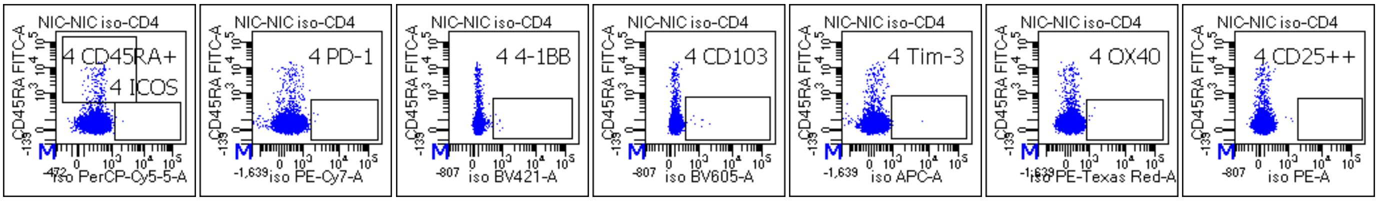

CD8+ T cells

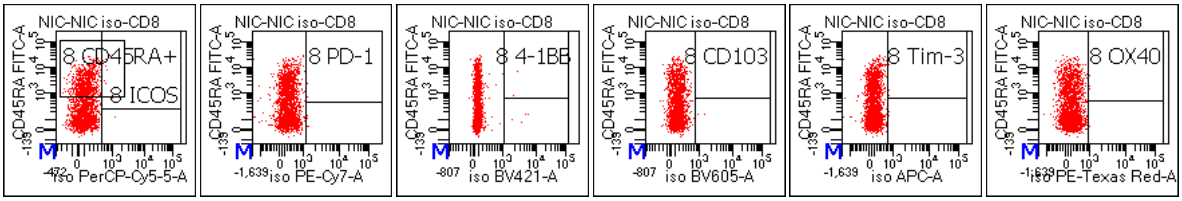

Tube: NIC

Population	#Events	%Parent	%Total
All Events	24,733	####	100.0
Live	20,104	81.3	81.3
CD45	19,499	97.0	78.8
Lym	13,944	71.5	56.4
singlet 1	13,929	99.9	56.3
singlet 2	13,852	99.4	56.0
CD3	12,598	90.9	50.9
CD4	9,871	78.4	39.9
4 CD45RA+	126	1.3	0.5
4 CD25++	581	5.9	2.3
4 ICOS	698	7.1	2.8
4 PD-1	8,230	83.4	33.3
4 4-1BB	157	1.6	0.6
4 CD103	6,889	69.8	27.9
4 Tim-3	329	3.3	1.3
4 OX40	680	6.9	2.7
CD8	2,178	17.3	8.8
8 ICOS	226	10.4	0.9
8 PD-1	1,559	71.6	6.3
8 4-1BB	13	0.6	0.1
8 CD103	1,148	52.7	4.6
8 Tim-3	259	11.9	1.0
8 OX40	25	1.1	0.1
8 CD45RA+	708	32.5	2.9
Mye	4,557	23.4	18.4

Tube: NIC iso

Population	#Events	%Parent	%Total
All Events	24,733	####	100.0
Live	20,110	81.3	81.3
CD45	19,490	96.9	78.8
Lym	13,937	71.5	56.3
singlet 1	13,917	99.9	56.3
singlet 2	13,855	99.6	56.0
CD3	12,651	91.3	51.2
CD4	9,862	78.0	39.9
4 CD45RA+	152	1.5	0.6
4 CD25++	0	0.0	0.0
4 ICOS	2	0.0	0.0
4 PD-1	3	0.0	0.0
4 4-1BB	6	0.1	0.0
4 CD103	9	0.1	0.0
4 Tim-3	4	0.0	0.0
4 OX40	5	0.1	0.0
CD8	2,190	17.3	8.9
8 ICOS	9	0.4	0.0
8 PD-1	6	0.3	0.0
8 4-1BB	7	0.3	0.0
8 CD103	2	0.1	0.0
8 Tim-3	5	0.2	0.0
8 OX40	4	0.2	0.0
8 CD45RA+	713	32.6	2.9
Mye	4,578	23.5	18.5

NIC Myeloid cells (RES29)

	PE CF594	BV510	BV605	BV711	BV786	Alexa Fluor 700	APC Cy7
NIC Myelo	CCR3	CD15	CD11b	CD33	CD45	CD14	Live/Dead

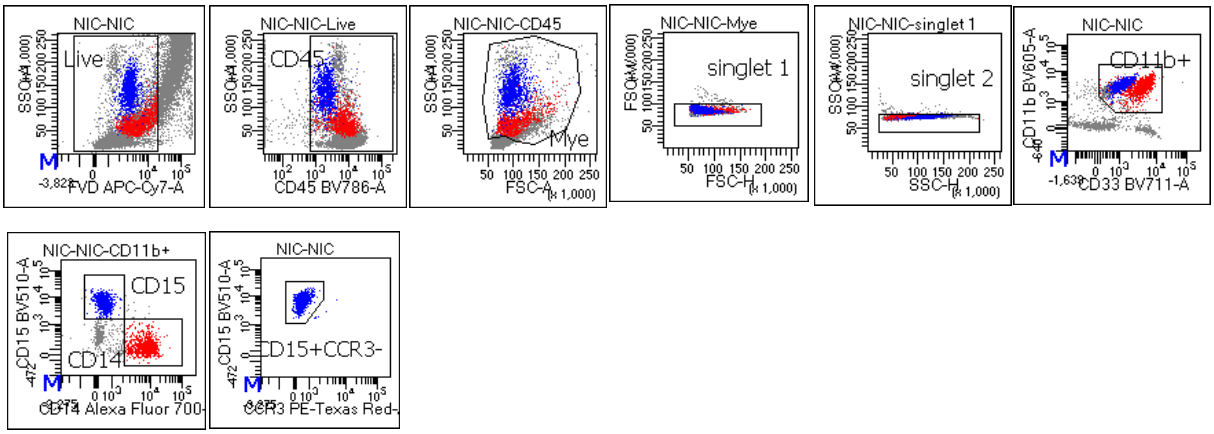

Tube: NIC

Population	#Events	%Parent	%Total
All Events	27,907	####	100.0
Live	21,156	75.8	75.8
CD45	19,760	93.4	70.8
Mye	3,328	16.8	11.9
singlet 1	3,276	98.4	11.7
singlet 2	3,219	98.3	11.5
CD11b+	2,337	72.6	8.4
CD14	961	41.1	3.4
CD15	945	40.4	3.4
CD15+CCR3-	939	99.4	3.4

PB lymphocytes (RES29)

	FITC	PerCP eFluor 710	PE	PE CF594	PE Cy7	BV421	BV510	BV605	BV711	APC	Alexa Fluor 700	APC Cy7
PB lympho	CD45RA	ICOS	CD25	OX40	PD-1	4-1BB	CD8	CD103	CD4	Tim-3	CD3	CD19
PB lympho iso	CD45RA	iso	iso	iso	iso	iso	CD8	iso	CD4	iso	CD3	CD19

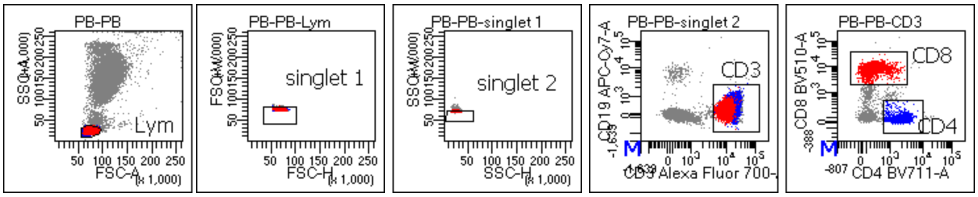

CD4+ T cells

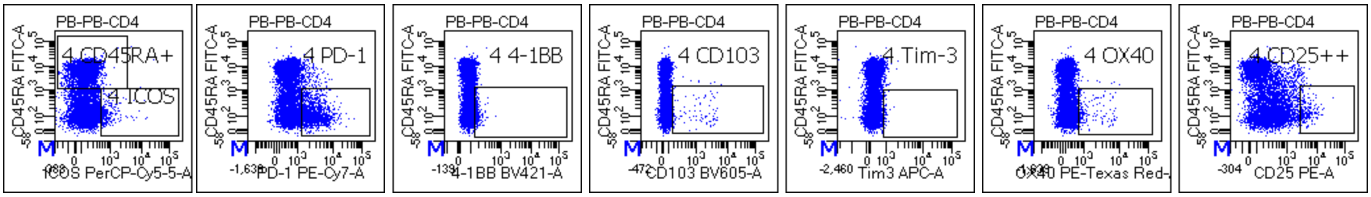

CD8+ T cells

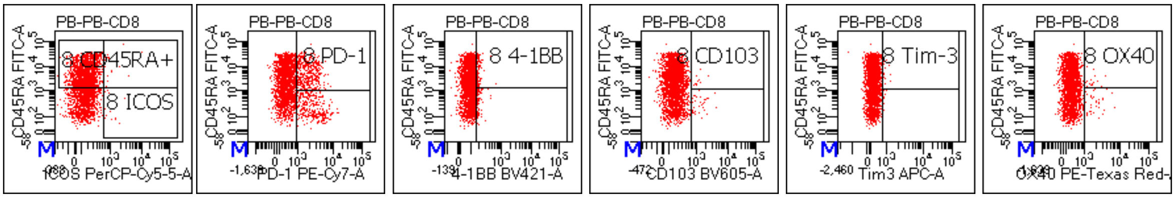

PB lymphocyte isotype-control (RES29)

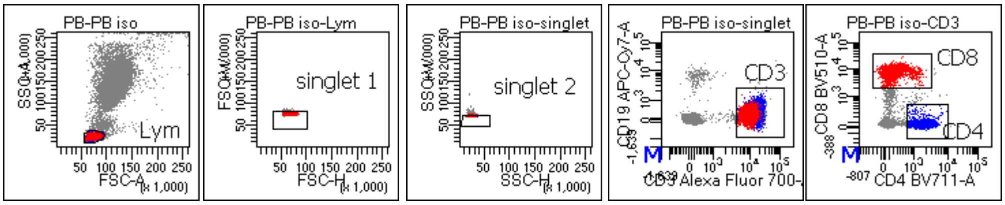

CD4+ T cells

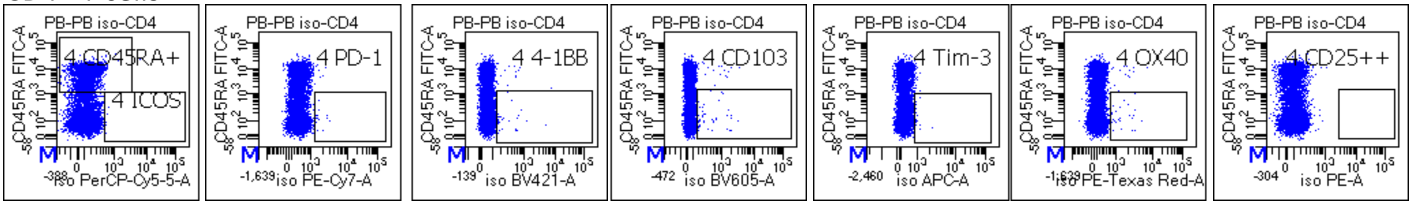

CD8+ T cells

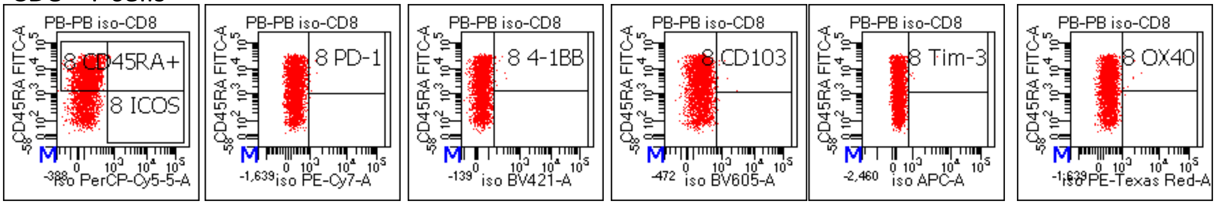

Tube: PB

Population	#Events	%Parent	%Total
All Events	30,690	####	100.0
Lym	13,203	43.0	43.0
singlet 1	13,189	99.9	43.0
singlet 2	13,093	99.3	42.7
CD3	10,313	78.8	33.6
CD4	6,361	61.7	20.7
4 CD45RA+	2,546	40.0	8.3
4 ICOS	239	3.8	0.8
4 Tim-3	56	0.9	0.2
4 PD-1	1,275	20.0	4.2
4 4-1BB	238	3.7	0.8
4 CD103	85	1.3	0.3
4 CD25++	201	3.2	0.7
4 OX40	143	2.2	0.5
CD8	2,628	25.5	8.6
8 CD45RA+	1,551	59.0	5.1
8 ICOS	35	1.3	0.1
8 Tim-3	29	1.1	0.1
8 PD-1	742	28.2	2.4
8 4-1BB	176	6.7	0.6
8 CD103	44	1.7	0.1
8 OX40	52	2.0	0.2

Tube: PB iso

Population	#Events	%Parent	%Total
All Events	31,169	####	100.0
Lym	13,515	43.4	43.4
singlet 1	13,503	99.9	43.3
singlet 2	13,418	99.4	43.0
CD3	10,550	78.6	33.8
CD4	6,288	59.6	20.2
4 CD45RA+	2,380	37.8	7.6
4 ICOS	6	0.1	0.0
4 Tim-3	25	0.4	0.1
4 PD-1	18	0.3	0.1
4 4-1BB	29	0.5	0.1
4 CD103	44	0.7	0.1
4 CD25++	0	0.0	0.0
4 OX40	26	0.4	0.1
CD8	2,916	27.6	9.4
8 CD45RA+	1,714	58.8	5.5
8 ICOS	5	0.2	0.0
8 Tim-3	7	0.2	0.0
8 PD-1	14	0.5	0.0
8 4-1BB	11	0.4	0.0
8 CD103	16	0.5	0.1
8 OX40	10	0.3	0.0

Supplementary Figure S12. Gating strategy of the FACS analysis.
The gating strategy of the FACS analysis for tumor-infiltrating cells (TIC), normal lung-infiltrating cells (NIC), and peripheral blood (PB) was shown using BD LSRFortessa with FACSDiva software.

Supplementary Figure S14

TIC (RES20)

	FITC	PE	BV510	BV711	Alexa Fluor 700	APC Cy7
TIC	CD45RA	IFNγ	CD8	CD4	CD3	Live/Dead
TIC isotype	CD45RA	iso	CD8	CD4	CD3	Live/Dead

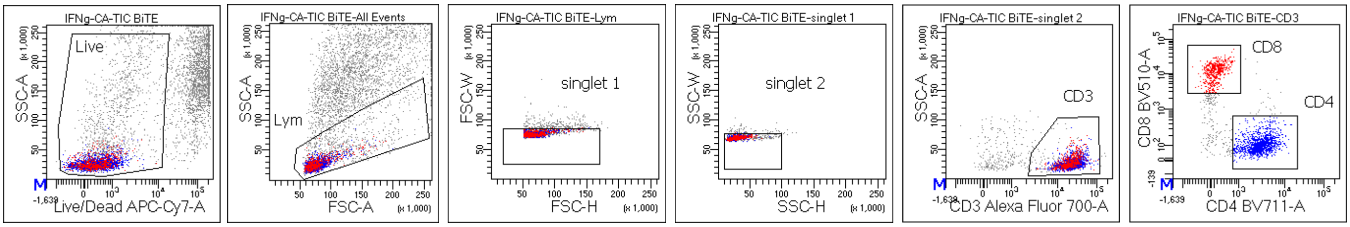

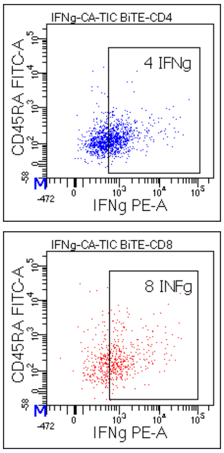

Tube: TIC BiTE

Population	#Events	%Parent	%Total
All Events	5,514	####	100.0
Live	3,039	55.1	55.1
Lym	2,550	83.9	46.2
singlet 1	2,312	90.7	41.9
singlet 2	2,244	97.1	40.7
CD3	1,979	88.2	35.9
CD4	1,213	61.3	22.0
4 IFNg	660	54.4	12.0
CD8	560	28.3	10.2
8 INFg	335	59.8	6.1

TIC isotype-control (RES20)

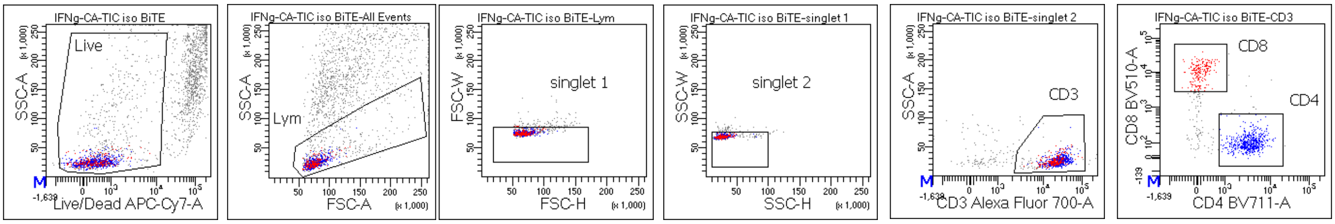

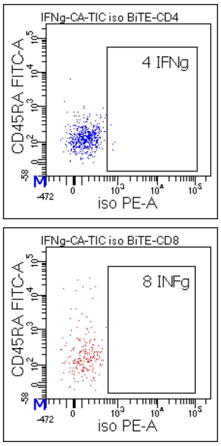

Tube: TIC iso BiTE

Population	#Events	%Parent	%Total
All Events	2,328	####	100.0
Live	1,265	54.3	54.3
Lym	1,094	86.5	47.0
singlet 1	1,016	92.9	43.6
singlet 2	973	95.8	41.8
CD3	859	88.3	36.9
CD4	552	64.3	23.7
4 IFNg	4	0.7	0.2
CD8	210	24.4	9.0
8 INFg	0	0.0	0.0

Supplementary Figure S13. Gating strategy of the IFNg secretion assay. The gating strategy of the IFNg secretion assay for tumor-infiltrating cells (TIC) was shown using BD LSRFortessa with FACSDiva software.
